# Agomelatine as adjunctive therapy with SSRIs or SNRIs for major depressive disorder: a multicentre, double-blind, randomized, placebo-controlled trial

**DOI:** 10.1186/s12916-025-03951-0

**Published:** 2025-03-05

**Authors:** Yumeng Ju, Wenwen Ou, Haoran Chen, Limin Yang, Yan Long, Hui Liang, Zhenman Xi, Mei Huang, Wentao Chen, Guanyi Lv, Fangzhou Shao, Bangshan Liu, Jin Liu, Zexuan Li, Mei Liao, Weiye Liang, Zhijian Yao, Yan Zhang, Lingjiang Li

**Affiliations:** 1 Department of Psychiatry, and National Clinical Research Center for Mental Disorders, The Second Xiangya Hospital of Central South University, Changsha, Hunan 410011 China; 2 Mental Health Institute of Central South University, China National Technology Institute On Mental Disorders, Hunan Technology Institute of Psychiatry, Hunan Key Laboratory of Psychiatry and Mental Health, Changsha, Hunan 410011 China; 3 Department of Psychiatry, Wenzhou Seventh People’s Hospital, Wenzhou, Zhejiang 325000 China; 4 Department of Psychiatry, Shandong Mental Health Center, Shandong University, Jinan, Shandong 250000 China; 5 Department of Psychiatry, Zhuzhou Third Hospital, Zhuzhou, Hunan 412000 China; 6 Peking University Huilongguan Clinical Medical School, Beijing Huilongguan Hospital, Changping District, Beijing 100096 China; 7 Department of Psychiatry, Affiliated Nanjing Brain Hospital, Nanjing Medical University, Nanjing, Jiangsu 210000 China

**Keywords:** Agomelatine, Adjunctive therapy, Major depressive disorder, RCT

## Abstract

**Background:**

In general, traditional antidepressants often have limited efficacy in patients with major depressive disorder (MDD). Agomelatine, as an antidepressant with a different mechanism of action, might have adjunctive effects on traditional antidepressants. This study aimed to investigate the augmentation effect of agomelatine versus placebo in treating MDD patients who failed to respond to selective serotonin reuptake inhibitors (SSRIs) and serotonin-noradrenaline reuptake inhibitors (SNRIs).

**Methods:**

This is an 8-week, multi-centred, double-blinded, randomized, and placebo-controlled trial. Participants diagnosed with MDD and demonstrated inadequate response to SSRI or SNRI lasting at least 2 weeks were randomly allocated to receive either agomelatine or placebo in conjunction with SSRIs or SNRIs. The 17 items of the Hamilton Depression Scale (HAMD-17) were employed to assess depression severity. The primary outcome is the total score of HAMD-17 at week 8. Secondary outcomes included HAMD-17 scores at weeks 2 and 4 and clinical remission and response over 8 weeks. Adverse events (AEs) reported in both groups were recorded. A linear mixed model was established for both primary and secondary outcomes.

**Results:**

A total of 123 eligible participants were included, among which 60 were randomized into the agomelatine group, and 63 were randomized into the placebo group. The between-group difference in HAMD-17 score reduction from baseline to week 8 was not significant (difference = − 0.12, 95% CI = − 3.94 to 3.70, *P* = 0.90; Cohen’s *d* = 0.022). In addition, we did not observe significant differences between the two treatment groups for secondary outcomes, including response remission, and AEs.

**Conclusions:**

This study did not obtain significant findings in favour of the augmentation effect of agomelation for MDD patients. However, agomelatine was generally well tolerated and demonstrated a favourable safety profile when used in combination with SSRIs and SNRIs.

Trial registration.

This trial is registered at ClinicalTrials.gov (https://clinicaltrials.gov), the registration number is NCT 04589143.

**Supplementary Information:**

The online version contains supplementary material available at 10.1186/s12916-025-03951-0.

## Background

Major depressive disorder (MDD) is a prevalent and debilitating mental health condition characterized by persistent feelings of sadness, hopelessness, and a lack of interest or pleasure in daily activities [1]. While it significantly impacts an individual’s emotional well-being, cognitions, and behaviours, leading to a range of functional impairments [2], it also comes along with increased mortality, morbidity and medical cost, imposing a significant burden to not only MDD patients themselves, but also their families and the society [3]. In general, pharmacotherapy with antidepressant drugs is often considered the first-line treatment for MDD patients, which also remains to be one of the mainstays of treatments throughout the course of MDD [4–6]. Among these, selective serotonin reuptake inhibitors (SSRIs) and serotonin-noradrenaline reuptake inhibitors (SNRIs) are the most well-known and commonly used antidepressants. However, more than half of MDD patients do not adequately respond to antidepressants at adequate doses after 8 weeks of treatment [7].

To date, there is limited evidence to guide the management of MDD patients who have taken adequate doses of SSRIs or SNRIs but do not demonstrate clinically meaningful improvements in their depressive symptoms. Yet, the Canadian Network for Mood and Anxiety Treatments (CANMAT) [8] has recommended that psychiatric practitioners and clinicians reconsider treatment if patients do not show a response to the prescribed antidepressant after 2–4 weeks, such as switching antidepressants or adding adjunctive drugs to augment the efficacy of the existing antidepressant [9]. Cleare and colleagues [10] have pointed out that an augmentation regimen, which is to add adjunctive medication, was better than switching the existing antidepressants*.* Aligned with such findings, a previous systematic review focusing on adjunctive treatments for MDD has ascertained that several drugs, such as cariprazine, aripiprazole, risperidone, olanzapine, quetiapine and ziprasidone, are effective in terms of reducing depressive symptoms among MDD patients who have an inadequate response to antidepressants in the previous treatment [11, 12]. Of note, cariprazine, aripiprazole, quetiapine, and brexpiprazole are the only four antipsychotic drugs endorsed by the U.S. Food and Drug Administration (FDA) for adjunctive treatment of MDD patients [12]. Yet, an augmentation regimen with antipsychotic medications is often accompanied by more adverse events, such as extrapyramidal symptoms, as well as less tolerability [13], which necessitates further research into alternative treatment options.

In recent years, several studies have hypothesized that adding another antidepressant with a mechanism of action to traditional SSRIs and SNRIs could potentially have a complementary effect that booster the effect of SSRIs or SNRIs [6]. Thus, agomelatine, an antidepressant of which the mechanism of action differs from the classical SSRIs and SNRIs, has increasingly captured the attention of researchers. To date, agomelatine has been recognized for several advantages. First, agomelatine exhibits melatonergic agonist activity due to its higher affinity for the MT1 and MT2 receptors [14], potentially facilitating the regulation of circadian rhythms and improving sleep patterns [15]. Second, agomelatine is a receptor antagonist at the 5-HT2b and 5-HT2c receptors [16, 17 ]. Through antagonizing the 5-HT2c receptor, it promotes the release of dopamine and norepinephrine from the prefrontal cortex but has no effect on extracellular serotonin levels [18]. Given that agomelatine has been found to promote the release of dopamine, it might have potential benefits in terms of improving some typical symptoms among depressed patients, such as anhedonia [19], which is largely associated with the level of dopamine and dopamine-related rewarding system [20],[21]. Meanwhile, the release of norepinephrine could potentially improve patients’ anxiety symptoms [22]. Last, due to the unique receptor binding profile of agomelatine, it is rarely associated with withdrawal symptoms following abrupt discontinuation [18]. To summarize, all the above evidence suggested that agomelatine could potentially benefit MDD patients in their antidepressant treatments.

Up to now, approximately 10 randomized controlled trials (RCT) support the efficacy of agomelatine as monotherapy for depression [23–29]. Additionally, RCTs have shown that agomelatine has advantages in reducing anxiety symptoms and improving sleep in patients with depression [23]. Regarding safety and acceptability, although agomelatine has certain side effects, such as liver dysfunction, increasing evidence supports its high tolerance and acceptability when treating depressed patients [30]. Moreover, a previous network meta-analysis has demonstrated a higher acceptability of agomelatine compared to other antidepressants [31]. Given the unique mechanism of agomelatine and its well-established tolerability and acceptability, the use of agomelatine in addition to other antidepressants may improve the overall efficacy for MDD. Yet, no clinical trials have thoroughly focused on exploring the augmentative effects of agomelatine for other antidepressants applied in MDD treatments.

The current study aims to investigate the efficacy and safety of augmenting antidepressant treatment with agomelatine for patients with MDD who did not demonstrate satisfying responses to SSRIs and SNRIs during their early phase of treatment; this study also aims to explore the effects of augmenting antidepressant treatment with agomelatine on various aspects, including anxiety, anhedonia, sleep quality, social functioning, and cognitive function in patients with MDD. Given the multiple pharmacological effects of agomelatine, we hypothesize that agomelatine is more efficacious than a placebo control.

## Methods

### Study design and participants

This study was an 8-week multi-centred, double-blind, randomized, placebo-controlled clinical trial. From October 1st, 2020, to January 30th, 2023, participants were recruited from six hospitals, including the Second Xiangya Hospital of Central South University, Beijing Huilongguan Hospital, Nanjing Brain Hospital, Wenzhou Mental Health Center, Shandong Mental Health Center, and the Third Hospital of Zhuzhou. The target population of this study were patients with depression who exhibited inadequate response to their initial treatment with SSRIs or SNRIs. The study procedures were approved by the medical ethics committees of all six hospitals and were conducted in accordance with the Consolidated Standards Of Reporting Trials (CONSORT) guidelines (Additional File: Table 1). Written informed consent was obtained from all participants before enrollment (ClinicalTrials.gov: NCT 04589143).

The inclusion criteria included (1) female or male patients aged between 18 and 60 years; (2) meeting the diagnostic criteria of MDD as defined in the Diagnostic and Statistical Manual of Mental Disorder fifth edition (DSM-5); (3) currently in a depressive episode, and the course of the current episode is less than a year; (4) score greater 4 or higher on the Clinical Global Impression-Disease Severity (CGI-S) scale; and (5) having been treated with monotherapy using an SSRI (except fluvoxamine and paroxetine) or SNRI for more than 2 weeks in the current depressive episode, with a dosage greater or equal to the minimum effective dose. Minimum effective doses for the commonly used classes of antidepressants include sertraline: ≥ 50 mg; fluoxetine: ≥ 20 mg; citalopram: ≥ 20 mg; escitalopram: ≥ 10 mg; venlafaxine: ≥ 75 mg; duloxetine: ≥ 60 mg; (6) demonstrating an inadequate response to antidepressant treatment lasting at least 2 weeks. Inadequate response is defined as a less than 20% improvement in the scores on the 17 items of the Hamilton Depression Scale (HAMD-17) after a 2-week period of antidepressant treatment, while participants scored ≥ 17 in HAMD-17 before 2 weeks ago; or it could be defined as patient’s self-report that their depressive symptom improved by less than 20% after more than a 2-week period of antidepressant treatment, with their current HAMD-17 score ≥ 17; (7) a minimum of 6 years of education, with the ability to provide informed consent and to independently complete all scales and assessments; (8) agreement from primary healthcare providers and patients to maintain current antidepressant treatment during the 8-week follow-up period.

Besides, if patients have met one or more of the following criteria, they are considered ineligible for participation: (1) meeting other DSM-5 diagnostic criteria for psychiatric disorders, such as generalized anxiety disorder, schizophrenia, bipolar disorder, or mental disorders related to alcohol and drug dependence; (2) current severe suicidal ideations or suicide attempts; (3) depressive disorder with psychotic symptoms; (4) having been treated with antidepressants in combination with other psychiatric medications (small doses of benzodiazepines are allowed); (5) having received anticoagulants (e.g. heparin, warfarin), glucocorticoids, or treatment for thyroid diseases in the past 3 months; (6) having received other non-medication treatments in the past six months, such as repetitive transcranial magnetic stimulus (rTMS), or systematic psychotherapy for more than 10 times; (7) having received any neurocognitive assessment similar to this study in the past 12 months; (8) current or previous history of brain organic diseases or loss of consciousness for more than 5 min; (9) current or previous history of major physical diseases (including rheumatic immune system diseases, endocrine and metabolic diseases, and nervous system diseases); (10) pregnancy or lactation in women; (11) current or previous history of seizures; (12) colour blindness (which would hinder neurocognitive testing); (13) positive urine drug screening results or abnormal thyroid function test; (14) liver function tests showing transaminase (alanine aminotransferase (ALT) and aspartate aminotransferase (AST)) levels that are 2 times above the upper limit of the normal range; (15) abnormal results from electrocardiograms (ECG) (QTc ≥ 430 ms for males and ≥ 450 ms for females).

Participants were recruited at the study sites after being approached by researchers while receiving their treatment or through advertising materials (i.e. flyers and posters) that had been approved by the institutional ethics committees. To ascertain eligibility, on-site screening and clinical laboratory tests were conducted 1–5 days prior to randomization. All participants provide written informed consent at least 24 h prior to inclusion.

### Randomization and masking

The participants who were eligible and gave written informed consent were randomized to take either 25–50 mg of agomelatine or an identical placebo group. Randomization and the allocation of the participant to either the agomelatine-augmented therapy group or the placebo-therapy group was performed by the drug administrator (HM, LBS, and LJ) using computer-generated blocks with a size of 4. The randomization was stratified by the types of antidepressants the participants have been on, either SSRIs or SNRIs. Allocation concealment was achieved by drug packs with identification codes, which were randomly generated to ensure that agomelatine and placebo packs were indistinguishable. Allocation details for each participant were securely stored in sealed, opaque envelopes and sent to each site along with the drug packs. Placebos were identical to agomelatine in size, weight, shape, and colour. The drug administrator had no further role in the rest of the trial. Participants and study personnel remained blinded to participants’ treatment allocation until after the database was locked.

### Assessment

The clinical raters at each site were in charge of participant recruitment and assessment. Before the study, the clinical raters (JYM, ZJ, CHR, YLM, LY, CWT, SFZ, and LGY) have had the inter-rater consistency training. All clinical raters were not involved in the randomization and were blinded to the allocation of eligible participants.

Clinical raters at each study site recorded the patients’ depressive symptoms at baseline and at the end of week 2, week 4, and week 8. Depression severity was assessed by HAMD-17 [32], and the higher total scores of HAMD-17 indicated severe depressive symptoms. Additional measures were recorded at the same time point, these measures are detailed in Additional File: Additional Efficacy Measures [33–40]. Meanwhile, the process of assessment is also presented in Additional File: Table 2.

### Treatment procedure

Patients who met the inclusion criteria and have provided informed consent were included in the study. Once enrolled in the study, the participant must maintain a stable dose of their SSRIs or SNRIs treatment until the end of the trial or the time they request to quit the study. Each participant was randomized to either 25–50 mg of agomelatine, or an identical placebo. The initial dosage of augmentation drugs (either agomelatine or placebo) was 25 mg/day, which was supported by evidence from previous clinical trials, and has demonstrated a relatively satisfactory effect[16]. For dosage adjustment, clinicians may adjust the dosage according to the participants’ condition. Some participants maintained their dosages at 25 mg/day, and the rest increased the current dosage of augmentation drugs to 50 mg/day by the end of week 2, and the dosage will be maintained until the end of the trial. To ensure that patients were following the prescribed dosing schedule, we collected the patients’ empty pill boxes at each follow-up visit.

### Primary and secondary outcomes

The primary outcome was the total scores of HAMD-17 at week 8 (trial end). Secondary outcomes were HAMD-17 scores at week 2 and week 4. The scores of 9-items Patient Health Questionnaire (PHQ-9), Hamilton Anxiety Scale (HAMA), 7-tiem Generalized Anxiety Disorder Scale (GAD-7), Clinical Global Impression Severity (CGIS), Snaith-Hamilton Pleasure Scale (SHAPS), Athens Insomnia Scale (AIS), Sheehan Disability Scale (SDS), cognitive functions, clinical remission (defined as HAMD-17 scores ≤ 7) [32], and clinical response (defined as a score reduction on the HAMD-17 ≥ 50%) [41] at week 8 were also recognized as additional efficacy outcomes.

### Satefy outcomes

For safety outcomes, we analysed scores from the Side Effect Rating Scale (SERS) at week 8. During the treatment phase, adverse events (AEs) were also assessed by directly asking participants about them. Any potential AEs were then assessed and recorded by the raters on the same day. Routine blood tests, assessments of liver and kidney function, electrolyte levels, as well as electrocardiograms, were conducted as part of the regular monitoring protocol at baseline, week 2, week 4 and week 8.

### Statistical analysis

We aimed to recruit 60 participants in each group, giving 80% power to detect a standardized effect size (Cohen’s *d*) of 0.52 at a two-sided 5% significance level. This would correspond to a difference of 3 points on the HAMD-17 between groups, which is considered the minimal important difference in depression research [42]. Allowing for a 10% loss to follow-up, we planned to recruit 67 participants in each group.

Data analyses were performed in the Statistical Package for the Social Sciences (SPSS) 26.0 and R package lmer4 (https://cran.rproject.org/web/packages/lme4/). Data distribution was determined by probability plot. For baseline demographic and clinical characteristics, continuous variables were presented as means and standard deviations, while categorical variables were reported as frequencies and percentages within each category. Continuous variables were compared using an independent sample *t*-test, and categorical variables were compared using Pearson’s chi-square test or Fisher’s exact test between different groups.

Missing values during follow-up were imputed by the last observation carried forward procedure (LOCF). All analyses of primary and secondary outcomes were conducted with the intention­to­treat (ITT) sample (i.e. all patients randomly assigned to a treatment group who met the inclusion criteria and did not withdraw consent after treatment initiation). For the primary and continuous secondary outcomes, linear mixed effects models (LMM) with random intercepts for centres and fixed effects for the treatment group and baseline scores of the respective outcome variables were calculated. Estimated differences were calculated to assess treatment in influencing the outcome of interest. Cohen’s *d* was calculated as an effect size. For response and remission rates, logistic regression models with random intercepts for centres and fixed effects for baseline HAMD-17 total scores were used. Odds ratios were calculated to assess treatment effects. Numbers needed to treat (NNT) were used to calculate effect sizes. The NNT for a binary outcome comparing treatments A versus B denotes the number of participants required to be treated with A to observe one additional response/remission compared to the same number of participants treated with B.

An exploratory analysis was carried out to assess the robustness of our primary analysis. First, confounding factors such as age, gender, education, depressive episode and the overall course of depression were included as fixed variables in the LMM to detect their influence on the results. Additionally, the type of antidepressants (SSRI/SNRI), recruitment assessment methods (according to self-evaluation criteria/according to observer evaluation criteria), or the dose of agomelatine (25 mg/ 50 mg) were included as random variables to control their effect. Moreover, to explore the impact of initial antidepressant dosage on the augmentation effect of agomelatine, we converted the antidepressant dosages to fluoxetine 20 mg equivalents, as described by previous research [43, 44]. Subsequent analysis of the original dataset, without employing LOCF, was conducted to avoid the impact of data imputation.

### Role of the funding source

The funder of the study had no role in study design, data collection, data analysis, data interpretation, or writing of the report.

## Results

**Fig. 1:**
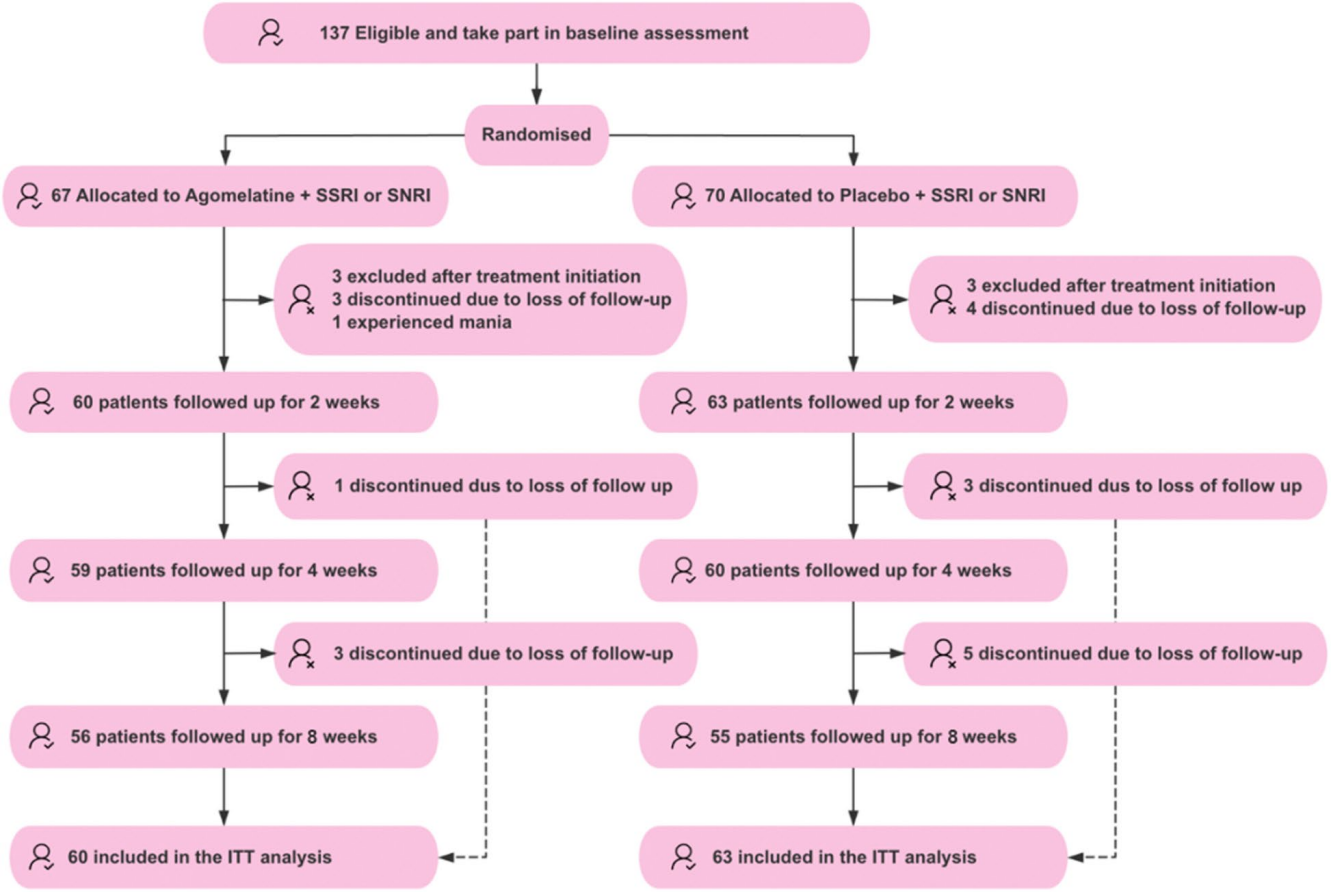
Flowchart of the randomized controlled trial. ITT, intention-to-treat

A total of 137 eligible participants were enrolled in the study, with 67 receiving agomelatine augmenting treatment, and 70 receiving placebo treatment. In the agomelatine group, 3 participants were excluded, 3 participants were discontinued due to loss of follow-up, and 1 participant quit due to experience of mania by the end of week 2. Subsequently, 1 participant and 3 participants were discontinued due to loss of follow-up at weeks 4 and 8, respectively. In the placebo group, 3 participants were excluded and 4 participants were discontinued by week 2. Subsequently, 3 participants and 5 participants discontinued due to loss of follow-up at week 4 and week 8, respectively. Ultimately, 123 participants were included in the ITT analysis, among which 60 participants received agomelatine augmenting treatment, and 63 participants received placebo, giving 80% power to detect a standardized effect size (Cohen’s *d*) of 0.51 at a two-sided 5% significance level. The overall procedure of participant enrolment and follow-up is shown in Fig. 1. Additionally, the detailed information regarding the antidepressant taken by the patients is presented in Additional File: Table 3–4.

The demographic and clinical profiles of the patients are detailed in Table 1. The mean ages of participants were 26.6 (SD 7.96) in the placebo-controlled group and 25.0 (SD 6.90) in the agomelatine group. In addition, the mean score on the HAMD-17 at baseline was 19.9 (SD 3.9) in the agomelatine group and 20.9 (SD 4.9) in the placebo-controlled group. Notably, there were no notable differences between the two groups concerning all the demographic and clinical characteristics during baseline.

**Table 1:** Baseline demographic and clinical characteristics

Variables	Agomelatine + SSRI or SNRI (*N* = 60)	Placebo + SSRI or SNRI (*N* = 63)	*P*-value
**Age (years)**	25.0 (6.90)	26.6 (7.96)	0.216^a^
**Gender**			0.561^b^
Male	24 (40.0%)	22 (34.9%)	
Female	36 (60.0%)	41 (65.1%)	
**Education**			0.827^b^
Junior high and below	5 (8.3%)	7 (11.1%)	
Senior high and vocational	18 (30.0%)	20 (31.7%)	
Bachelor and above	37 (61.7%)	36 (57.1%)	
**BMI**	21.2 (3.75)	21.2 (3.03)	0.962^a^
**Marital status**			0.669^b^
Married	49 (81.7%)	48 (76.2%)	
Single	9 (15.0%)	11 (17.5%)	
Divorced or widowed	2 (3.3%)	4 (6.3%)	
**Number of episodes**			0.354^b^
1	27 (45.0%)	28 (44.4%)	
2	15 (25.0%)	22 (34.9%)	
3	18 (30.0%)	13 (20.6%)	
> 3	27 (45.0%)	28 (44.4%)	
**Overall course of disease (month)**	7 (12, 45)	6 (10, 24)	0.063^c^
**Antidepressant**			0.715^b^
SSRIs	44 (73.3%)	48 (76.2%)	
SNRIs	16 (26.7%)	15 (23.8%)	
**Baseline HAMD-17**	19.9 (3.9)	20.9 (4.9)	0.204^a^
**Baseline HAMA**	17.4 (5.9)	17.7 (5.5)	0.798^a^
**Baseline PHQ-9**	15.8 (4.8)	16.1 (4.9)	0.695^a^
**Baseline GAD-7**	9.8 (4.6)	8.5 (4.9)	0.154^a^
**Baseline CGI-S**	4.8 (0.7)	4.6 (0.8)	0.463^a^
**Baseline SHAPS**	34.8 (6.8)	36.5 (5.9)	0.127^a^
**Baseline AIS**	12.0 (4.3)	12.7 (3.9)	0.398^a^
**Baseline SDS**	6.7 (2.4)	6.8 (2.1)	0.795^a^
**Executive function**			
DSB	6.3 (2.2)	6.4(1.9)	0.625^a^
Stroop Color-Word	42.8 (16.9)	47.2 (14.6)	0.154^a^
TMT-B	63.3 (21.4)	58.2 (24.3)	0.276^a^
DSST	63.5 (14.1)	60.5 (15.1)	0.301^a^
**Attention**			
DSF	8.6 (1.7)	8.9 (1.7)	0.303^a^
Stroop-Word	90.4 (19.2)	90.1 (18.9)	0.930^a^
**Processing speed**			
TMT-A	26.0 (7.2)	28.5 (9.0)	0.125^a^
Stroop-Color	76.1 (20.7)	75.8 (18.9)	0.935^a^
**Memory**			
HVLT-R	26.8 (4.8)	25.5 (5.3)	0.209^a^
HVLT-delayed recall trial	9.5 (2.1)	9.3 (3.4)	0.845^a^

Data are *n* (%) or mean (SD). *BMI *Body mass index, *HAMD-17 *Hamilton Depression Rating Scale 17-item version, *HAMA *Hamilton Anxiety Rating Scale, *PHQ-9 *9-item Patient Health Questionnaire-9, *GAD-7 *7-tiem Generalized Anxiety Disorder Scale, *CGI-S *Clinical Global Impression Severity Scale, *SHAPS *Snaith Hamilton Anhedonia Pleasure Scale, *AIS *Athens Insomnia Scale, *SDS *Sheehan Disability Scale, *EQ-5D-3L *EuroQol Five Dimensions Questionnaire Three-Level, *DSB *Digit Span Backward Test, *TMT-A *Trial–Making Part A task, *TMT-B *Trial–Making Part B test, *DSF *Digit Span Forward test, *HVLT *Hopkins Verbal Learning Test, *SSRI *Selective serotonin reuptake inhibitor, *SNRI *Serotonin-noradrenaline reuptake inhibitor

^a^ *P* values obtained by independent-sample *t* test

^b^ *P* values obtained by Pearson’s *χ* ^2^ test

^c^ *P* values obtained by Mann–Whitney *U* test

The efficacy of treatment (agomelatine compared to placebo) on the primary outcome (HAMD-17 score) at week 8 was not significant (Table 2). At the trial end, the mean scores on HAMD-17 were 7.2 (SD 5.2) in the agomelatine group and 7.4 (SD 6.3) in the placebo group, and the adjusted difference in means was − 0.12 (95% CI − 3.94 to 3.70, *P* = 0.90; Cohen’s *d* = − 0.022), indicating no significant difference between groups (Table 2).

**Table 2:** Primary outcomes, secondary outcomes and safety

Variables	Agomelatine + SSRI or SNRI		Placebo + SSRI or SNRI		Comparison		
	No	Mean (SD)	No	Mean (SD)	Adjusted difference in means (95% CI)	*P* value	Effect size (Cohen’s *d*)
**Primary outcome**							
HAMD-17 at week 8	60	7.2 (5.2)	63	7.4 (6.3)	− 0.12 (− 3.94 to 3.70)	0.90	− 0.022
**Secondary outcomes**							
Remission	60	30 (50.0%)	63	33 (52.3%)	0.88 (0.42–1.85) ^a^	0.74	41 ^b^
Response	60	36 (60.0%)	63	41 (65.2%)	0.85 (0.40–1.80) ^a^	0.68	20^b^
HAMD-17 at week 2	60	11.7 (4.7)	63	12.4 (6.8)	− 0.20 (− 4.12 to 3.73)	0.83	− 0.038
HAMD-17 at week 4	60	8.5 (5.2)	63	9.1 (5.6)	− 0.27 (− 4.31 to 3.78)	0.76	− 0.057
PHQ-9	60	7.3 (4.8)	63	7.0 (6.2)	0.39 (− 2.79 to 3.57)	0.67	0.077
HAMA	60	7.0 (6.0)	63	6.8 (6.9)	0.36 (− 3.16 to 3.88)	0.72	0.066
GAD-7	60	4.8 (4.1)	63	3.2 (3.7)	1.32 (− 0.39 to 3.03)	0.06	0.351
AIS	60	4.7 (3.8)	63	4.8 (5.2)	− 0.02 (− 2.48 to 2.44)	0.98	− 0.004
CGI severity	60	2.5 (1.4)	63	2.3 (1.5)	0.16 (− 0.95 to 1.28)	0.46	0.134
SHAPS	60	29.2 (8.3)	63	29.1 (8.5)	0.87 (− 4.66 to 6.40)	0.51	0.122
SDS	56	2.6 (2.1)	56	2.3 (2.3)	0.36 (− 0.94 to 1.65)	0.36	0.176
**Executive function**							
DSB	51	6.9 (2.2)	48	7.2 (1.8)	− 0.09 (− 0.81 to 0.63)	0.76	− 0.063
DSST	45	68.9 (12.3)	43	69.4 (14.2)	− 2.47 (− 8.28 to 3.33)	0.25	− 0.251
Stroop Color-Word	49	52.8 (17.5)	48	55.9 (18.8)	− 1.61 (− 14.62 to 11.39)	0.59	− 0.113
TMT-B	42	45.8 (17.6)	45	47.7 (21.6)	− 2.43 (− 21.28 to 16.41)	0.52	− 0.142
**Attention**							
DSF	50	9.0 (1.6)	48	9.3 (1.8)	0.03 (− 0.52 to 0.57)	0.91	0.024
Stroop-Word	49	94.1 (16.6)	48	96.8 (17.1)	− 2.88 (− 11.38 to 5.63)	0.39	− 0.176
**Processing speed**							
TMT-A	42	21.6 (6.6)	45	21.3 (7.3)	1.59 (− 2.16 to 5.34)	0.26	0.253
Stroop-Color	49	80.8 (18.9)	48	85.5 (20.0)	− 5.36 (− 15.95 to 5.23)	0.13	− 0.332
**Memory**							
HVLT-R	48	25.7 (6.4)	50	25.9 (5.3)	1.39 (− 2.4 to 5.19)	0.68	− 0.086
HVLT-delayed recall trial	48	9.6 (3.8)	50	8.8 (2.4)	0.77 (− 0.70 to 2.25)	0.20	0.264
**SERS**	60	0.5 (1.2)	63	0.4 (1.2)	− 0.01 (− 0.54 to 0.52)	0.95	− 0.012

a, estimated OR and the 95%CI were calculated in logistic LMM; b, effect sizes were calculated as NNT. *HAMD-17 *Hamilton Depression Rating Scale 17-item version, *HAMA *Hamilton Anxiety Rating Scale, *PHQ-9 *9-item Patient Health Questionnaire-9, *GAD-7 *7-tiem Generalized Anxiety Disorder Scale, *CGI-S *Clinical Global Impression Severity Scale, *SHAPS *Snaith Hamilton Anhedonia Pleasure Scale, *AIS *Athens Insomnia Scale, *SDS *Sheehan Disability Scale, *EQ-5D-3L *EuroQol Five Dimensions Questionnaire Three-Level, *DSB *Digit Span Backward Test, *DSST *Digital Symbol Substitution test, *TMT-A *Trial–Making Part A task, *TMT-B *Trial–Making Part B test, *DSF *Digit Span Forward test, *HVLT *Hopkins Verbal Learning Test, *SERS *Side Effects Rating Scale

**Fig. 2:**
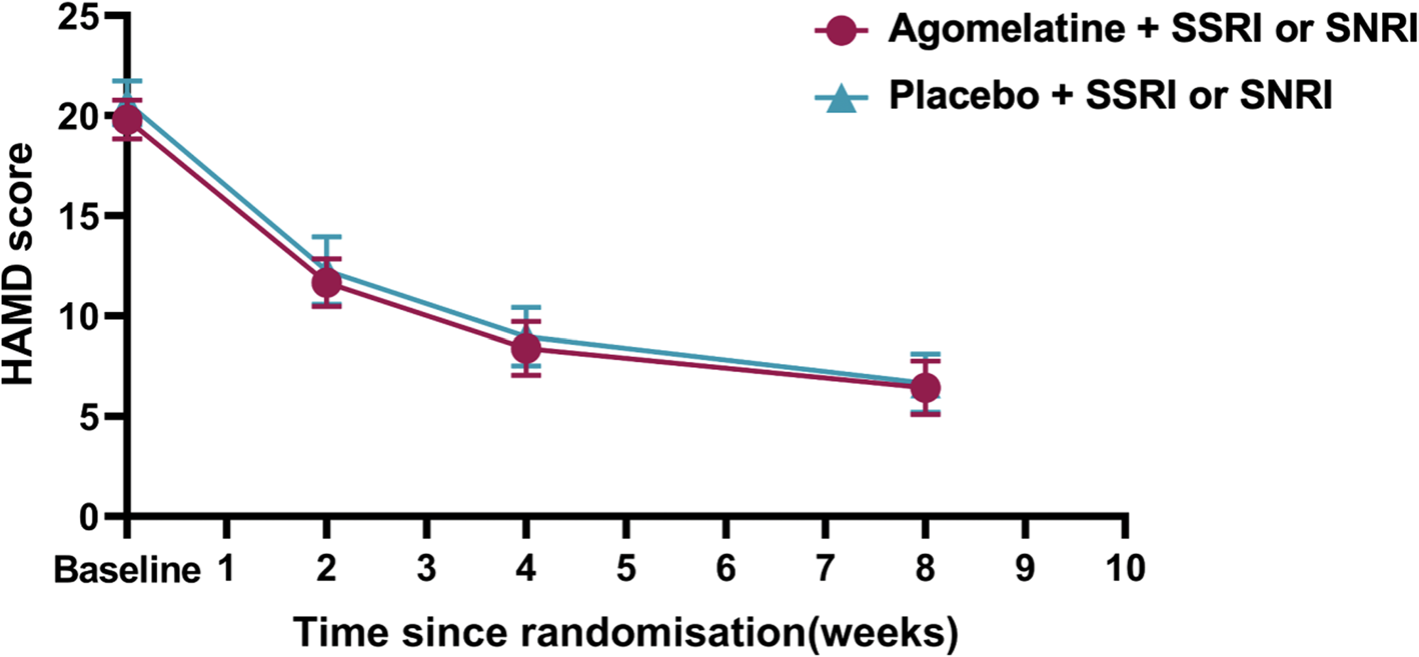
Change on HAMD-17 from baseline to week 8

To evaluate whether agomelatine can expedite early treatment and shorten recovery time, we analysed the effect of treatment on HAMD-17 scores at week 2 and week 4. However, no significant differences were observed between treatment groups in terms of HAMD-17 score. The data distribution and changes in HAMD-17 are displayed in Fig. 2.

In terms of the secondary outcomes, no significant differences were observed between the two treatment groups (Table 2). In particular, no significant effect of agomelatine was observed on PHQ-9, HAMA, GAD-7, CGI-S, SDS, AIS, SHAPS and all the domains in cognitive function (Table 2). At week 8, 36 participants (60.0%) receiving agomelatine responded to treatment and 30 (50.0%) achieved remission. In the placebo group, 41 patients (65.2%) were response and 33 patients (52.3%) achieved remission (Table 2). The effect of agomelatine compared to placebo on the remission and response over the course of 8 weeks of observation was also non-significant.

In the sensitivity analysis, the LMM analysis demonstrated that confounding factors such as age, gender, and education exerted minimal influence on the results (Additional File: Table 5). In addition, the type of antidepressant (SSRI/SNRI), recruitment assessment methods (according to self-evaluation criteria/according to observer evaluation criteria), or the dose of agomelatine (25 mg/50 mg) also did not exhibit a statistically significant effect on the primary and secondary outcomes (Additional File: Table 6–8). The initial dosage of antidepressants (SSRI/SNRI) did not show a statistically significant effect on the primary and secondary outcomes. However, the social functioning, as measured by SDS, showed greater improvement in the agomelatine group compared to the placebo group (estimated difference = 0.94, 95% CI − 0.39 to 2.28, *P* = 0.02, Cohen’s *d* = 0.512, Additional File: Table 9). Subsequent analysis of the original dataset, without employing LOCF, showed that the treatment did not yield significant effects on the results (Additional File: Table 10).

No drug-related serious adverse events occurred during the trial. There is also no significant effect of agomelatine augmentation on the SERS scores at week 8 (Table 2). AEs were reported in 14 out of 60 patients (23.3%) in the agomelatine group and 17 out of 63 patients (26.9%) in the placebo group (Additional File: Table 11). No significant abnormalities were observed in terms of liver function, electrolyte levels, and electrocardiograms (Additional File: Table 12). Meanwhile, other minor adverse effects of agomelatine, including drowsiness, dizziness, headache, fatigue, and gastrointestinal symptoms, were reported in 1.67% to 10% of patients in the treatment group (Additional File: Table 13). There appears to be minimal correlation between these adverse events and the intervention.

## Discussion

To the best of our knowledge, the current study is the first RCT that investigates the efficacy of agomelatine as an augmentation treatment for MDD patients when maintaining their SSRIs or SNRIs treatment as usual. We found no evidence to support the augmentation effect of agomelatine in alleviating depressive symptoms in patients who remained depressed following at least two weeks of antidepressant treatment. Additionally, agomelatine did not show a statistically significant effect on secondary outcomes, including the remission and response rates among patients, as well as the patient’s self-rated depression, anxiety, anhedonia, sleep quality, social functioning and cognitive functioning. Power analysis conducted prior to the study indicated that a sample size of 60 per group would provide 80% power to detect a medium effect size (Cohen’s d = 0.52). However, these results did not meet the thresholds for statistical significance for any of the primary or secondary outcomes. In terms of safety and tolerability, only a few adverse events were reported in both treatment groups, while the placebo and agomelatine groups did not significantly differ on safety and tolerability measures. All of which indicated that agomelatine, as an adjunctive drug for SSRIs or SNRIs, is not an optimal choice for enhancing the efficacy of antidepressant treatments.

For the primary analysis, improvements in the scores of HAMD-17 from baseline to trial end (week 8) were not significantly different between the agomelatine group and placebo group. Additionally, our research also failed to demonstrate its effectiveness in terms of improving sleep and reducing symptoms of anxiety or anhedonia [19],[22],[15]. Although it has been suggested from a pharmacodynamic perspective that the agonism of agomelatine on both types of melatonergic receptors MT_1_ and MT_2_ and antagonism of serotonergic 5-HT_2C_ receptors could transfer into a synergistic effect for antidepressant purposes [18],[45], results from the current analysis elucidated that agomelatine failed to reflect the pharmacological mechanism of its augmenting effects.

In fact, numerous studies have been dedicated to exploring the potentiating effects of agomelatine in antidepressant therapy. Previous case reports aimed to enhance the effectiveness of treatment for depression and achieve earlier remissions by combining agomelatine with other antidepressants. However, there is no consensus [46, 47]. In particular, as reported in individual cases, when using agomelatine in addition to venlafaxine, it could facilitate better control over depressive symptoms without detrimental effects on patient’s physical functioning, such as live function and blood pressure [47]. Nevertheless, evidence-based conclusions regarding the efficacy of using adjunctive drugs are still limited. Aligned with previous findings, our study further demonstrated that the combination of antidepressants has limited efficacy in terms of the improvement in clinical outcomes for MDD patients [6]. To delve into the underlying reasons, many other factors might impact the results. Since each combination might have a different pharmacodynamic mechanism of action, the combinations of agomelatine with different antidepressants might result in different interactions between medications, which would further lead to variations in terms of their efficacy and tolerability [45]. In the current study, we did not limit the specific type of SSRIs or SNRIs that participants were prescribed, which led to different treatment regimens with agomelatine, and this combination of different regimens might result in different interactions between different medications that may limit the synergistic efficacy of agomelatine.

For the secondary analysis, relatively small differences between the agomelatine and placebo groups were observed at the trial end (week 8), but the 95% confidence intervals surrounding the difference between groups comprised the null for most secondary outcomes. As to the acceptability and safety, the agomelatine group did not significantly differ from the placebo group, and there were only a few all-cause discontinuation cases and adverse events were reported in both groups. In particular, 7 out of 67 participants (10.4%) in the agomelatine group discontinued their participation in the study during the follow-up period, while 1 participant (1.4%) quit the study due to the experience of mania. Whereas in the placebo group, a total of 12 participants (17.9%) discontinued their participation due to loss of follow-up. In short, the above-mentioned statistics suggested a relatively satisfactory adherence and a rate of adverse events in the intervention group, implying a good acceptability and safety of agomelatine.

It is noteworthy that approximately half of the participants who were instructed to take agomelatine achieved remission by the end of week 8, and such, a remission rate is relatively higher than the statistics reported in previous research, in which the remission rates ranged from 16 to 30% [27, 29]. One possible explanation for an improved remission rate in the current study might be the participants’ depression severity at baseline. More specifically, participants in the previous research started the treatments with more severe depression [27, 29]. As reported in their baseline assessments, their average HAMD scores were approximately ranged from 26 to 28, which indicated severe depression among participants in the previous research. In the current study, our participants scored approximately 20 on HAMD-17 at baseline, indicating moderate depression. With such a different baseline characteristic of participants, it might be inferred that patients with less severe depression are more likely to achieve remission after treatments. Meanwhile, participants in previous research were accompanied by a higher level of anxiety [29] as compared to participants in the current research, which might impede participants’ progress to achieve remission. On the other hand, this study is a clinical trial in which participants could obtain medications for free. Given that the prognosis of depression is associated with several socioeconomic factors, including patients’ employment status and financial strain [48], free access to antidepressant medications potentially alleviated the financial burden on patients and their families, which could also be recognized as a protective psychosocial factor. Additionally, easier access to care and the structured trial environment with regular follow-ups may have improved treatment adherence and outcomes, creating conditions that differ from routine clinical practice. Therefore, these factors may limit the generalizability of our findings.

### Strength and limitations

Several strengths are worth mentioning in the current study. The participants, the clinicians and the administrators were all blinded to the allocation of the treatment conditions. The drop-out rates and the follow-up rates were considerably satisfactory throughout the trial at all study sites. To assess the impact of missing data on the primary analysis, a sensitivity analysis was conducted. In addition, whether the missing data were estimated under the assumption of a best- or worst-case scenario or using multiple imputations, the observed difference in HAMD-17 scores and other outcome measures at the trial end between agomelatine and placebo groups were small.

Our findings should also be interpreted considering several limitations. First, we recruited 137 participants at baseline, with only 123 included in the final analysis. This small sample size may limit our ability to detect the true effect of agomelatine in augmenting patients' previous antidepressant treatments. Second, this study primarily included patients with inadequate responses to 2 weeks of SSRI or SNRI treatment. Moreover, the 8-week duration of this RCT may have been insufficient to assess the long-term efficacy and safety of agomelatine as an adjunctive therapy. Third, this study did not standardize concomitant treatment to better reflect real-world clinical practices, where augmentation strategies often involve SSRIs and SNRIs. The sensitivity analysis indicated that the type of antidepressants did not affect the primary outcomes, but the initial dosage may have some impact on the secondary outcome of social functioning. However, this finding is limited by the small sample size. Future studies with a more balanced allocation or a stratified design could better isolate agomelatine’s effects and reduce variability. Fourth, due to dosage adjustments during follow-up, two augmentation dosage groups (25 mg/day and 50 mg/day) were included. This dosing variability may have influenced the assessment of agomelatine's efficacy, though sensitivity analysis showed no significant difference between the two groups. Finally, plasma concentration testing can determine if agomelatine has reached an effective therapeutic dose. Future studies could assess adherence using plasma concentration monitoring.

## Conclusions

To conclude, limited benefits of agomelatine for augmenting SSRIs or SNRIs were observed in the current research, which suggested that agomelatine shall not yet be recommended as an adjunctive drug for MDD patients as a routine intervention strategy in primary care for those who remain depressed after adequate treatment with SSRIs or SNRIs antidepressants. However, agomelatine has many benefits and advantages as the previous study ascertained, given that many limitations were observed in the current study, which warrants future studies to investigate antidepressant augmentation regimens with agomelatine.

## Supplementary Information

Additional file 1: Table 1. The CONsolidated Standards Of Reporting Trialsguideines. Table 2. List of Study Measures and Data Collection Schedule. Additional Efficacy Measures: This section in the additional file contains detailed measures and assessments applied in the study. Table 3. Current Antidepressant by Treatment Group. Table 4. Dosage of the initial antidepressant prescribed to each patient and their corresponding conversions into fluoxetine 20mg equivalents. Table 5. The impact of confounding factors on the augmenting antidepressant treatment with agomelatine. Table 6. The impact of the type of antidepressanton the augmenting antidepressant treatment with agomelatine. Table 7. The impact of recruitment assessment methods on the augmenting antidepressant treatment with agomelatine. Table 8. The impact of different dose of agomelatineon the augmenting antidepressant treatment with Agomelatine. Table 9. The impact of different antidepressantson the effect of augmenting antidepressant treatment with agomelatine. Table 10. The efficacy of augmenting antidepressant treatment with agomelatine without employing LOCF. Table 11. A comparison of adverse event between the two treatment groups over the course of 8 weeks. Table 12. A comparison of abnormal transaminase levels between the two treatment groups over the course of 8 weeks. Table 13. A comparison of adverse event between the two treatment groups at week 8

## Data Availability

After the publication of this article, de-identified individual patient data will be available for non-commercial academic projects that have a valid research question and a clearly defined hypothesis. Data can be requested from the corresponding author. All requests will be reviewed by ZY and LLJ to ensure the stated requirements are met. If approved, researchers will be required to sign a data access agreement, which includes obtaining approval from an institutional ethics board for the project. The de-identified datasets and data dictionary will then be provided by the authors through a secure data transfer service.

## Abbreviations

MDD: Major depressive disorder
SSRIs: Selective serotonin reuptake inhibitors
SNRIs: Serotonin-noradrenaline reuptake inhibitors
HAMD-17: 17-Item of Hamilton Depression Scale
AEs: Adverse events
CANMAT: Canadian Network for Mood and Anxiety Treatments
FDA: U.S. Food and Drug Administration
RCT: Randomized controlled trials
CONSORT: Consolidated Standards Of Reporting Trials
DSM-5: Diagnostic and Statistical Manual of Mental Disorder fifth edition
CGI-S: Clinical Global Impression-Disease Severity
rTMS: Repetitive transcranial magnetic stimulus
ALT: Alanine aminotransferase
AST: Aspartate aminotransferase
ECG: Electrocardiograms
PHQ-9: 9-Item Patient Health Questionnaire
HAMA: Hamilton Anxiety Scale
GAD-7: 7-Tiem Generalized Anxiety Disorder Scale
SHAPS: Snaith-Hamilton Pleasure Scale
AIS: Athens Insomnia Scale
SDS: Sheehan Disability Scale
SERS: Side Effect Rating Scale
SPSS: Statistical Package for the Social Sciences
ITT: Intention-to-treat
LMM: Linear mixed effects models
LOCF: Last observation carried forward
NNT: Numbers needed to treat
EQ-5D-3L: EuroQol Five Dimensions Questionnaire Three-Level
DSB: Digit Span Backward Test
TMT-A: Trial-Making Part A task
TMT-B: Trial-Making Part B test
DSF: Digit Span Forward test
HVLT: Hopkins Verbal Learning Test
SD: Standard deviation
