# Supplementary material for: Agomelatine as adjunctive therapy with SSRIs or SNRIs for major depressive disorder: a multicentre, double-blind, randomized, placebo-controlled trial
Source: BMC Med. 2025 Mar 5;23:137. doi: 10.1186/s12916-025-03951-0 (PMC11881269; doi:10.1186/s12916-025-03951-0)
Supplement: Supplementary file 1 — Additional file 1: Table 1. The CONsolidated Standards Of Reporting Trialsguideines. Table 2. List of Study Measures and Data Collection Schedule. Additional Efficacy Measures: This section in the additional file contains detailed measures and assessments applied in the study. Table 3. Current Antidepressant by Treatment Group. Table 4. Dosage of the initial antidepressant prescribed to each patient and their corresponding conversions into fluoxetine 20mg equivalents. Table 5. The impact of confounding factors on the augmenting antidepressant treatment with agomelatine. Table 6. The impact of the type of antidepressanton the augmenting antidepressant treatment with agomelatine. Table 7. The impact of recruitment assessment methods on the augmenting antidepressant treatment with agomelatine. Table 8. The impact of different dose of agomelatineon the augmenting antidepressant treatment with Agomelatine. Table 9. The impact of different antidepressantson the effect of augmenting antidepressant treatment with agomelatine. Table 10. The efficacy of augmenting antidepressant treatment with agomelatine without employing LOCF. Table 11. A comparison of adverse event between the two treatment groups over the course of 8 weeks. Table 12. A comparison of abnormal transaminase levels between the two treatment groups over the course of 8 weeks. Table 13. A comparison of adverse event between the two treatment groups at week 8 [file 12916_2025_3951_MOESM1_ESM.docx]

Table 1. The CONsolidated Standards Of Reporting Trials(CONSORT) guideines

| Section/Topic | Item No | Checklist item | Reported on page No |
| --- | --- | --- | --- |
| Title and abstract | | | |
|  | 1a | Identification as a randomised trial in the title | 1 |
|  | 1b | Structured summary of trial design, methods, results, and conclusions (for specific guidance see CONSORT for abstracts) | 2-3 |
| Introduction | | | |
| Background and objectives | 2a | Scientific background and explanation of rationale | 4-6 |
|  | 2b | Specific objectives or hypotheses | 7 |
| Methods | | | |
| Trial design | 3a | Description of trial design (such as parallel, factorial) including allocation ratio | 7 |
|  | 3b | Important changes to methods after trial commencement (such as eligibility criteria), with reasons |  |
| Participants | 4a | Eligibility criteria for participants | 8 |
|  | 4b | Settings and locations where the data were collected | 7 |
| Interventions | 5 | The interventions for each group with sufficient details to allow replication, including how and when they were actually administered | 9-10 |
| Outcomes | 6a | Completely defined pre-specified primary and secondary outcome measures, including how and when they were assessed | 11-12 |
|  | 6b | Any changes to trial outcomes after the trial commenced, with reasons |  |
| Sample size | 7a | How sample size was determined | 12-13 |
|  | 7b | When applicable, explanation of any interim analyses and stopping guidelines | 14 |
| Randomisation: |  |  |  |
| Sequence generation | 8a | Method used to generate the random allocation sequence | 10 |
|  | 8b | Type of randomisation; details of any restriction (such as blocking and block size) | 10 |
| Allocation concealment mechanism | 9 | Mechanism used to implement the random allocation sequence (such as sequentially numbered containers), describing any steps taken to conceal the sequence until interventions were assigned | 10 |
| Implementation | 10 | Who generated the random allocation sequence, who enrolled participants, and who assigned participants to interventions | 10 |
| Blinding | 11a | If done, who was blinded after assignment to interventions (for example, participants, care providers, those assessing outcomes) and how | 10 |
|  | 11b | If relevant, description of the similarity of interventions | 10 |
| Statistical methods | 12a | Statistical methods used to compare groups for primary and secondary outcomes | 13 |
|  | 12b | Methods for additional analyses, such as subgroup analyses and adjusted analyses | 14 |
| Results | | | |
| Participant flow (a diagram is strongly recommended) | 13a | For each group, the numbers of participants who were randomly assigned, received intended treatment, and were analysed for the primary outcome | 14-15 |
|  | 13b | For each group, losses and exclusions after randomisation, together with reasons | 14-15 |
| Recruitment | 14a | Dates defining the periods of recruitment and follow-up | 14-15 |
|  | 14b | Why the trial ended or was stopped | 14-15 |
| Baseline data | 15 | A table showing baseline demographic and clinical characteristics for each group | 16-17 |
| Numbers analysed | 16 | For each group, number of participants (denominator) included in each analysis and whether the analysis was by original assigned groups | 14 |
| Outcomes and estimation | 17a | For each primary and secondary outcome, results for each group, and the estimated effect size and its precision (such as 95% confidence interval) | 17-18,20-21 |
|  | 17b | For binary outcomes, presentation of both absolute and relative effect sizes is recommended | 18,20-21 |
| Ancillary analyses | 18 | Results of any other analyses performed, including subgroup analyses and adjusted analyses, distinguishing pre-specified from exploratory | 18 |
| Harms | 19 | All important harms or unintended effects in each group (for specific guidance see CONSORT for harms) | 18-19 |
| Discussion | | | |
| Limitations | 20 | Trial limitations, addressing sources of potential bias, imprecision, and, if relevant, multiplicity of analyses | 25-26 |
| Generalisability | 21 | Generalisability (external validity, applicability) of the trial findings | 25-26 |
| Interpretation | 22 | Interpretation consistent with results, balancing benefits and harms, and considering other relevant evidence | 22-25 |
| Other information | | |  |
| Registration | 23 | Registration number and name of trial registry | 7 |
| Protocol | 24 | Where the full trial protocol can be accessed, if available |  |
| Funding | 25 | Sources of funding and other support (such as supply of drugs), role of funders | 29 |

| **Table 2. List of Study Measures and Data Collection Schedule** | | | | | | | | | | | | | | | | |
| --- | --- | --- | --- | --- | --- | --- | --- | --- | --- | --- | --- | --- | --- | --- | --- | --- |
| **Measures** | **Descriptions** | | | | | | **Data Collection Schedule** | | | | | | |  |  |  |
|  |  |  |  |  |  |  | **Enrollment** | **Week 2** | | **Week 4** | | **Week 8** | | | |  |
| **1. Sociodemographic Measures** |  |  |  | | | |  |  | |  | |  | | | |  |
| Gender | Self-reported gender: female, male, other |  |  | | | | √ |  | |  | |  | | | |  |
| Age | Self-reported age: years since birth. |  |  | | | | √ |  | |  | |  | | | |  |
| Education | Self-reported educational attainment: none, primary, secondary, bachelor, postgraduate |  |  | | | | √ |  | |  | |  | | | |  |
| BMI | Calculated by using self-reported weight and height |  |  | | | | √ |  | |  | |  | | | |  |
| Marital Status | Self-reported marital status: yes / no. |  |  | | | | √ |  | |  | |  | | | |  |
| Number of episodes | As recorded |  |  | | | | √ |  | |  | |  | | | |  |
| Overall course of disease | As recorded |  |  | | | | √ |  | |  | |  | | | |  |
| Prescribed Antidepressants | Self-reported: SSRIs or SNRIs |  |  | | | | √ |  | |  | |  | | | |  |
| **2. Primary Outcome** |  |  |  | | | |  |  | |  | |  | | | |  |
| Depressive Symptoms | HAMD-17 at Week 8 |  |  | | | |  |  | |  | | √ | | | |  |
| **3. Secondary Outcomes** |  |  |  | | | |  |  | |  | |  | | | |  |
| Clinical Rmission | Defined as HAMD-17 scores ≤ 7 by Week 8 |  |  | | | |  |  | |  | | √ | | | |  |
| Clinical Response | Defined as a score reduction on the HAMD-17 ≥ 50% by Week 8 |  |  | | | |  |  | |  | | √ | | | |  |
| Depressive Symptoms during the 8-week trial | HAMD-17 at Week 2 |  |  | | | |  | √ | |  | |  | | | |  |
|  | HAMD-17 at Week 4 |  |  | | | |  |  | | √ | |  | | | |  |
| Self-reported depressive symptoms | Assessed by 9-item Patient Health Questionnaire (PHQ-9) |  |  | | | | √ | √ | | √ | | √ | | | |  |
| Anxiety symptoms | Assessed by Hamilton Anxiety Scale (HAMA) |  |  | | | | √ | √ | | √ | | √ | | | |  |
|  | Assessed by 7-item Generalized Anxiety Disorder Scale (GAD-7) |  |  | | | | √ | √ | | √ | | √ | | | |  |
| Sleep Quality | Assessed by Athens Insomnia Scale (AIS) |  |  | | | | √ | √ | | √ | | √ | | | |  |
| Severity of illness | Assessed by Clinical Global Impression Severity (CGI-S) |  |  | | | | √ | √ | | √ | | √ | | | |  |
| Anhedonia | Assessed by Snaith-Hamilton Pleasure Scale (SHAPS) |  |  | | | | √ | √ | | √ | | √ | | | |  |
| Social functioning | Assessed by Sheehan Disability Scale (SDS) |  |  | | | | √ | √ | | √ | | √ | | | |  |
| Cognitive functioning | A series of Neurocognitive Tests | Executive function | | | Digit Span Backward Test (DSB) from WAIS-R (correctly completed number of trails) | | √ | √ | |  | | √ | | | |  |
|  |  |  |  |  | Digital Symbol Substitution test (DSST) | | √ | √ | |  | | √ | | | |  |
|  |  |  |  |  | The color-word interference condition of the Stroop Test (total correct number) | | √ | √ | |  | | √ | | | |  |
|  |  |  |  |  | The Trail–Making Part B (time to completion) | | √ | √ | |  | | √ | | | |  |
|  |  | Attention | | | Digit Span Forward test (DSF) from the WAIS-R (correctly completed number of trails) | | √ | √ | |  | | √ | | | |  |
|  |  |  |  |  | Stroop Word Test (total correct number) | | √ | √ | |  | | √ | | | |  |
|  |  | Processing speed | | | Trail–Making Part A task (time to completion) | | √ | √ | |  | | √ | | | |  |
|  |  |  |  |  | Stroop Color Test (total correct number) | | √ | √ | |  | | √ | | | |  |
|  |  | Memory | | | The Hopkins Verbal Learning Test, revised (HVLT-R, total correct number) | | √ | √ | |  | | √ | | | |  |
|  |  |  |  |  | The Hopkins Verbal Learning Test-delayed recall trial | | √ | √ | |  | | √ | | | |  |
| **4. Safety Outcomes** |  | | |  | |  |  | |  | |  | |  | |  |  |
| Side effects | Assessed by Side Effect Rating Scale (SERS) | | |  | |  | √ | | √ | | √ | | √ | |  |  |
| Adeverse events | As recorded | | |  | |  | √ | | √ | | √ | | √ | |  |  |
| Physical health monitoring | Blood test | | |  | |  | √ | | √ | | √ | | √ | |  |  |
|  | Assessments of liver and kidney function | | |  | |  | √ | | √ | | √ | | √ | |  |  |
|  | Electrolyte levels | | |  | |  | √ | | √ | | √ | | √ | |  |  |
|  | Electrocardiograms | | |  | |  | √ | | √ | | √ | | √ | |  |  |

**Additional Efficacy Measures:**

Self-reported depressive symptoms were measured by 1) Patient Health Questionnaire-9 (PHQ-9)^1^. 2) Anxiety symptoms were measured by Hamilton-Anxiety (HAMA) and 7-tiem Generalized Anxiety Disorder Scale (GAD-7)^1,2^. 3) The severity of illness was measured by Clinical Global Impression Severity (CGI­S)^3^. 4) Anhedonia was measured by Snaith-Hamilton Pleasure Scale (SHAPS)^4^. 5) Sleep quality were measured by Athens Insomnia Scale (AIS)^5^. 6) Social functioning was measured by Sheehan Disability Scale (SDS)^6^. 7) Cognitive function was measured using a range of neurocognitive tests, which comprised executive function, attention, processing speed and memory^7^. The Digit Span Backward Test (DSB) from WAIS-R (correctly completed number of trails), the Digital Symbol Substitution test (DSST), the color-word interference condition of the Stroop Test (total correct number) and the Trail–Making Part B (time to completion) was used to assess executive function. The Stroop Word Test (total correct number) and the Digit Span Forward test (DSF) from the WAIS-R (correctly completed number of trails) were included for measuring attention. The Trail–Making Part A task (time to completion) and Stroop Color Test (total correct number) were applied to assess processing speed. The Hopkins Verbal Learning Test, revised (HVLT-R, total correct number) and the delayed recall subset of HVLT-R were used to measure memory performance^7,8^.

**Table 3. Current Antidepressant by Treatment Group**

| **Current Antidepressant** | **All Participants** | **Agomelatine + SSRI or SNRI** | **Placebo + SSRI or SNRI** |
| --- | --- | --- | --- |
| Citalopram | 2 | 0 | 2 |
| Duloxetine | 13 | 10 | 3 |
| Escitalopram | 53 | 24 | 29 |
| Fluoxetine | 11 | 6 | 5 |
| Sertraline | 27 | 13 | 14 |
| Venlafaxine | 17 | 7 | 10 |
| Total | 123 | 60 | 63 |

SSRI=selective serotonin reuptake inhibitor; SNRI=serotonin-noradrenaline reuptake inhibitor

**Table 4. Dosage of the initial antidepressant prescribed to each patient and their corresponding conversions into fluoxetine 20mg equivalents**

| Subject | group | Inital antidepressant | Inital dose (mg) | Dose equivalent to fluoxetine 20 mg |
| --- | --- | --- | --- | --- |
| CS-101 | 0 | Escitalopram | 20 | 44.4 |
| CS-102 | 1 | Sertraline | 50 | 20.28 |
| CS-103 | 1 | Sertraline | NA | NA |
| CS-104 | 0 | Escitalopram | 15 | 33.3 |
| CS-105 | 1 | Sertraline | 100 | 40.56 |
| CS-106 | 0 | Sertraline | 75 | 30.42 |
| CS-107 | 1 | Escitalopram | NA | NA |
| CS-108 | 0 | Sertraline | 75 | 30.42 |
| CS-109 | 1 | Escitalopram | NA | NA |
| CS-110 | 0 | Escitalopram | 10 | 22.2 |
| CS-111 | 0 | Escitalopram | 10 | 22.2 |
| CS-112 | 1 | Escitalopram | 15 | 33.3 |
| CS-113 | 1 | Escitalopram | 20 | 44.4 |
| CS-114 | 0 | Escitalopram | 10 | 22.2 |
| CS-115 | 0 | Escitalopram | 10 | 22.2 |
| CS-116 | 1 | Escitalopram | 10 | 22.2 |
| CS-117 | 0 | Sertraline | 50 | 20.28 |
| CJ-118 | 0 | Escitalopram | 10 | 22.2 |
| CS-201 | 0 | Venlafaxine | 150 | 40.17 |
| CS-202 | 1 | Duloxetine | 60 | 20 |
| CS-203 | 0 | Venlafaxine | NA | NA |
| CS-204 | 1 | Venlafaxine | 225 | 60.26 |
| CS-205 | 0 | Venlafaxine | 150 | 40.17 |
| CS-206 | 1 | Venlafaxine | NA | NA |
| CS-208 | 0 | Venlafaxine | NA | NA |
| NJ-101 | 0 | Escitalopram | 10 | 22.2 |
| NJ-102 | 1 | Escitalopram | NA | NA |
| NJ-103 | 0 | Sertraline | 200 | 81.12 |
| NJ-104 | 1 | Fluoxetine | 20 | 20 |
| NJ-105 | 0 | Sertraline | 150 | 60.84 |
| NJ-106 | 0 | Sertraline | 200 | 81.12 |
| NJ-107 | 1 | Escitalopram | 20 | 44.4 |
| NJ-108 | 1 | Escitalopram | 20 | 44.4 |
| NJ-109 | 0 | Escitalopram | 20 | 44.4 |
| NJ-110 | 1 | Escitalopram | NA | NA |
| NJ-111 | 1 | Fluoxetine | 20 | 20 |
| NJ-112 | 0 | Fluoxetine | 30 | 30 |
| NJ-113 | 0 | Fluoxetine | 20 | 20 |
| NJ-114 | 1 | Escitalopram | NA | NA |
| NJ-115 | 0 | Escitalopram | 20 | 44.4 |
| NJ-117 | 0 | Escitalopram | 20 | 44.4 |
| NJ-119 | 1 | Sertraline | 100 | 40.56 |
| NJ-120 | 1 | Sertraline | 150 | 60.84 |
| NJ-121 | 0 | Citalopram | 20 | 20 |
| NJ-122 | 1 | Escitalopram | NA | NA |
| NJ-123 | 1 | Escitalopram | NA | NA |
| NJ-124 | 0 | Escitalopram | 20 | 44.4 |
| NJ-125 | 1 | Escitalopram | NA | NA |
| NJ-126 | 1 | Sertraline | 100 | 40.56 |
| NJ-127 | 0 | Escitalopram | 20 | 44.4 |
| NJ-128 | 0 | Escitalopram | 20 | 44.4 |
| NJ-129 | 1 | Escitalopram | NA | NA |
| NJ-130 | 0 | Escitalopram | 20 | 44.4 |
| NJ-131 | 1 | Sertraline | NA | NA |
| NJ-133 | 1 | Escitalopram | 10 | 22.2 |
| NJ-135 | 0 | Escitalopram | 20 | 44.4 |
| NJ-137 | 0 | Sertraline | NA | NA |
| NJ-139 | 1 | Escitalopram | 20 | 44.4 |
| NJ-141 | 0 | Fluoxetine | 20 | 20 |
| NJ-140 | 1 | Sertraline | 50 | 20.28 |
| NJ-144 | 0 | Sertraline | NA | NA |
| NJ-145 | 1 | Escitalopram | NA | NA |
| NJ-146 | 1 | Escitalopram | NA | NA |
| NJ-147 | 0 | Sertraline | NA | NA |
| NJ-149 | 0 | Sertraline | NA | NA |
| NJ-150 | 1 | Escitalopram | NA | NA |
| NJ-151 | 0 | Sertraline | NA | NA |
| NJ-152 | 1 | Fluoxetine | 50 | 50 |
| NJ-153 | 0 | Sertraline | 200 | 81.12 |
| NJ-201 | 0 | Venlafaxine | 225 | 60.26 |
| NJ-202 | 1 | Venlafaxine | 225 | 60.26 |
| NJ-204 | 1 | Duloxetine | 40 | 13.3 |
| NJ-205 | 1 | Duloxetine | NA | NA |
| NJ-206 | 0 | Venlafaxine | 225 | 60.26 |
| NJ-207 | 1 | Venlafaxine | 150 | 40.17 |
| NJ-208 | 0 | Duloxetine | 60 | 20 |
| NJ-209 | 1 | Duloxetine | 60 | 20 |
| NJ-210 | 0 | Venlafaxine | 225 | 60.26 |
| BJ-101 | 1 | Escitalopram | 20 | 44.4 |
| BJ-102 | 0 | Escitalopram | 20 | 44.4 |
| BJ-103 | 0 | Sertraline | NA | NA |
| BJ-104 | 1 | Escitalopram | NA | NA |
| BJ-105 | 0 | Escitalopram | 15 | 33.3 |
| BJ-106 | 0 | Escitalopram | 20 | 44.4 |
| BJ-107 | 1 | Escitalopram | 20 | 44.4 |
| BJ-108 | 1 | Escitalopram | 15 | 33.3 |
| BJ-109 | 1 | Sertraline | 150 | 60.84 |
| BJ-110 | 0 | Escitalopram | 20 | 44.4 |
| BJ-201 | 1 | Duloxetine | 60 | 20 |
| BJ-204 | 1 | Venlafaxine | 150 | 40.17 |
| BJ-203 | 0 | Duloxetine | 60 | 20 |
| BJ-205 | 1 | Duloxetine | 60 | 20 |
| ZZ-101 | 1 | Sertraline | 150 | 60.84 |
| ZZ-102 | 1 | Fluoxetine | 40 | 40 |
| ZZ-103 | 0 | Fluoxetine | 40 | 40 |
| ZZ-104 | 0 | Escitalopram | 20 | 44.4 |
| ZZ-105 | 1 | Sertraline | 100 | 40.56 |
| ZZ-106 | 1 | Escitalopram | 10 | 22.2 |
| ZZ-201 | 1 | Venlafaxine | 150 | 40.17 |
| ZZ-202 | 0 | Venlafaxine | 225 | 60.26 |
| ZZ-203 | 0 | Venlafaxine | 150 | 40.17 |
| WZ-103 | 1 | Sertraline | 50 | 20.28 |
| WZ-104 | 0 | Fluoxetine | 40 | 40 |
| WZ-105 | 0 | Escitalopram | 10 | 22.2 |
| WZ-107 | 0 | Sertraline | 200 | 81.12 |
| WZ-109 | 0 | Escitalopram | NA | NA |
| WZ-110 | 1 | Sertraline | 50 | 20.28 |
| WZ-111 | 0 | Citalopram | 40 | 40 |
| WZ-113 | 0 | Escitalopram | 10 | 22.2 |
| WZ-201 | 1 | Venlafaxine | 150 | 40.17 |
| WZ-202 | 1 | Duloxetine | 60 | 20 |
| WZ-203 | 0 | Venlafaxine | 150 | 40.17 |
| JN-101 | 0 | Escitalopram | 20 | 44.4 |
| JN-102 | 0 | Escitalopram | 15 | 33.3 |
| JN-103 | 0 | Escitalopram | 20 | 44.4 |
| JN-105 | 1 | Fluoxetine | 20 | 60 |
| JN-106 | 0 | Escitalopram | 20 | 44.4 |
| JN-107 | 0 | Escitalopram | 20 | 44.4 |
| JN-110 | 1 | Fluoxetine | 60 | 60 |
| JN-201 | 0 | Duloxetine | 60 | 20 |
| JN-202 | 1 | Duloxetine | 60 | 20 |
| JN-203 | 1 | Duloxetine | 60 | 20 |
| JN-204 | 1 | Duloxetine | 60 | 20 |

Group 0 refers to Placebo + SSRI or SNRI, while Group 1 refers to Agomelatine + SSRI or

SNRI；NA, not applicable

**Table 5. The impact of confounding factors on the augmenting antidepressant treatment with agomelatine**

| **Variables** | **Agomelatine + SSRI or SNRI** | | **Placebo + SSRI or SNRI** | | **Comparison** | | |
| --- | --- | --- | --- | --- | --- | --- | --- |
|  | No | Mean (SD) | No | Mean (SD) | Adjusted difference in means (95% CI) | P value | Effect size (Cohen’s d) |
| **Primary outcome** | | | | | | | |
| HAMD-17 at 8 week | 60 | 7.2 (5.2) | 63 | 7.4 (6.3) | -0.27 (-4.30 to 3.76) | 0.80 | -0.049 |
| **Secondary outcomes** | | | | | | | |
| remission | 60 | 30 (50.0%) | 63 | 33 (52.3%) | 1.0 (0.45 to 2.21) ^a^ | 0.99 | 42 ^b^ |
| response | 60 | 36 (60.0%) | 63 | 41 (65.2%) | 0.99 (0.45 to 2.18) ^a^ | 0.98 | 20 ^b^ |
| HAMD-17 at 2 week | 60 | 11.7 (4.7) | 63 | 12.4 (6.8) | -0.23 (-4.58 to 4.12) | 0.81 | -0.045 |
| HAMD-17 at 4 week | 60 | 8.5 (5.2) | 63 | 9.1 (5.6) | -0.39 (-4.60 to 3.81) | 0.66 | -0.083 |
| PHQ-9 | 60 | 7.3 (4.8) | 63 | 7.0 (6.2) | -0.01 (-3.37 to 3.35) | 0.99 | -0.001 |
| HAMA | 60 | 7.0 (6.0) | 63 | 6.8 (6.9) | 0.27 (-3.49 to 4.02) | 0.79 | 0.049 |
| GAD-7 | 60 | 4.8 (4.1) | 63 | 3.2 (3.7) | 1.28 (-0.64 to 3.21) | 0.07 | 0.347 |
| AIS | 60 | 4.7 (3.8) | 63 | 4.8 (5.2) | -0.21 (-2.79 to 2.37) | 0.80 | -0.047 |
| CGI severity | 60 | 2.5 (1.4) | 63 | 2.3 (1.5) | 0.11 (-1.00 to 1.23) | 0.62 | 0.112 |
| SHAPS | 60 | 29.2 (8.3) | 63 | 29.1 (8.5) | 0.10 (-5.60 to 5.81) | 0.94 | 0.015 |
| SDS | 56 | 2.6 (2.1) | 56 | 2.3 (2.3) | 0.26 (-1.02 to 1.54) | 0.51 | 0.131 |
| **Executive function** |  |  |  |  |  |  |  |
| DSB | 51 | 6.9 (2.2) | 48 | 7.2 (1.8) | -0.09 (-1.15 to 0.96) | 0.77 | -0.064 |
| DSST | 45 | 68.9 (12.3) | 43 | 69.4 (14.2) | -2.27 (-9.17 to 4.63) | 0.31 | -0.229 |
| Stoop Color-Word | 49 | 52.8 (17.5) | 48 | 55.9 (18.8) | -1.80 (-18.16 to 14.57) | 0.56 | -0.128 |
| TMT-B | 42 | 45.8 (17.6) | 45 | 47.7 (21.6) | -3.57 (-29.95 to 22.81) | 0.35 | -0.217 |
| **Attention** |  |  |  |  |  |  |  |
| DSF | 50 | 9.0 (1.6) | 48 | 9.3 (1.8) | -0.01 (-0.87 to 0.85) | 0.97 | -0.008 |
| Stroop-Word | 49 | 94.1 (16.6) | 48 | 96.8 (17.1) | -4.06 (-16.16 to 8.05) | 0.23 | -0.25 |
| **Processing speed** |  |  |  |  |  |  |  |
| TMT-A | 42 | 21.6 (6.6) | 45 | 21.3 (7.3) | 1.78 (-6.53 to 10.09) | 0.22 | 0.292 |
| Stroop-Color | 49 | 80.8 (18.9) | 48 | 85.5 (20.0) | -6.41 (-21.27 to 8.46) | 0.06 | -0.413 |
| **Memory** |  |  |  |  |  |  |  |
| HVLT-R | 48 | 25.7 (6.4) | 50 | 25.9 (5.3) | -0.51 (-5.06 to 4.04) | 0.66 | -0.092 |
| HVLT-delayed recall trial | 48 | 9.6 (3.8) | 50 | 8.8 (2.4) | 0.42 (-1.81 to 2.65) | 0.50 | 0.144 |
| **SERS** | 60 | 0.5 (1.2) | 63 | 0.4 (1.2) | -0.02 (-0.59 to 0.55) | 0.92 | -0.020 |

a, estimated OR and the 95%CI were calculated in logistic LMM; b, Effect sizes were calculated as NNT; HAMD-17=Hamilton Depression Rating Scale 17-item version; HAMA=Hamilton Anxiety Rating Scale; PHQ-9=9-item Patient Health Questionnaire-9; GAD-7=7-tiem Generalized Anxiety Disorder Scale; CGI-S=Clinical Global Impression Severity Scale; SHAPS=Snaith Hamilton Anhedonia Pleasure Scale AIS= Athens Insomnia Scale; SDS= Sheehan Disability Scale; EQ-5D-3L=EuroQol Five Dimensions Questionnaire Three-Level; DSB=Digit Span Backward Test; DSST=Digital Symbol Substitution test; TMT-A=Trail–Making Part A task; TMT-B= Trail–Making Part B test; DSF=Digit Span Forward test; HVLT=Hopkins Verbal Learning Test; SERS= Side Effects Rating Scale.

**Table 6. The impact of the type of antidepressant (SSRI/SNRI) on the augmenting antidepressant treatment with agomelatine**

| **Variables** | **Agomelatine + SSRI or SNRI** | | **Placebo + SSRI or SNRI** | | **Comparison** | | |
| --- | --- | --- | --- | --- | --- | --- | --- |
|  | No | Mean (SD) | No | Mean (SD) | Adjusted difference in means (95% CI) | P value | Effect size (Cohen’s d) |
| **Primary outcome** | | | | | | | |
| HAMD-17 at 8 week | 60 | 7.2 (5.2) | 63 | 7.4 (6.3) | -0.12 (-4.71 to 4.47) | 0.90 | -0.022 |
| **Secondary outcomes** | | | | | | | |
| remission | 60 | 30 (50.0%) | 63 | 33 (52.3%) | 0.90 (0.29 to 2.76) ^a^ | 0.74 | 41 ^b^ |
| response | 60 | 36 (60.0%) | 63 | 41 (65.2%) | 0.85 (0.40 to 1.80) ^a^ | 0.85 | 20 ^b^ |
| HAMD-17 at 2 week | 60 | 11.7 (4.7) | 63 | 12.4 (6.8) | -0.47 (-5.23 to 4.29) | 0.76 | -0.092 |
| HAMD-17 at 4 week | 60 | 8.5 (5.2) | 63 | 9.1 (5.6) | -0.43 (-5.22 to 4.37) | 0.69 | -0.092 |
| PHQ-9 | 60 | 7.3 (4.8) | 63 | 7.0 (6.2) | 0.36 (-3.63 to 4.34) | 0.82 | 0.071 |
| HAMA | 60 | 7.0 (6.0) | 63 | 6.8 (6.9) | 0.36 (-4.01 to 4.72) | 0.72 | 0.066 |
| GAD-7 | 60 | 4.8 (4.1) | 63 | 3.2 (3.7) | 1.32 (-1.2 to 3.84) | 0.06 | 0.351 |
| AIS | 60 | 4.7 (3.8) | 63 | 4.8 (5.2) | -0.08 (-3.32 to 3.15) | 0.95 | -0.019 |
| CGI severity | 60 | 2.5 (1.4) | 63 | 2.3 (1.5) | 0.16 (-1.09 to 1.42) | 0.46 | 0.134 |
| SHAPS | 60 | 29.2 (8.3) | 63 | 29.1 (8.5) | 0.11 (-7.65 to 7.87) | 0.96 | 0.016 |
| SDS | 56 | 2.6 (2.1) | 56 | 2.3 (2.3) | 0.34 (-1.60 to 2.27) | 0.40 | 0.169 |
| **Executive function** |  |  |  |  |  |  |  |
| DSB | 51 | 6.9 (2.2) | 48 | 7.2 (1.8) | -0.09 (-1.21 to 1.03) | 0.76 | -0.063 |
| DSST | 45 | 68.9 (12.3) | 43 | 69.4 (14.2) | -2.47 (-10.95 to 6.00) | 0.25 | -0.251 |
| Stoop Color-Word | 49 | 52.8 (17.5) | 48 | 55.9 (18.8) | -1.61 (-16.82 to 13.59) | 0.59 | -0.113 |
| TMT-B | 42 | 45.8 (17.6) | 45 | 47.7 (21.6) | -2.43 (-23.8 to 18.93) | 0.52 | -0.142 |
| **Attention** |  |  |  |  |  |  |  |
| DSF | 50 | 9.0 (1.6) | 48 | 9.3 (1.8) | 0.31 (-0.82 to 1.44) | 0.72 | 0.295 |
| Stroop-Word | 49 | 94.1 (16.6) | 48 | 96.8 (17.1) | -1.59 (-14.57 to 11.40) | 0.82 | -0.098 |
| **Processing speed** |  |  |  |  |  |  |  |
| TMT-A | 42 | 21.6 (6.6) | 45 | 21.3 (7.3) | 0.58 (-5.31 to 6.48) | 0.87 | 0.094 |
| Stroop-Color | 49 | 80.8 (18.9) | 48 | 85.5 (20.0) | -2.45 (-19.77 to 14.87) | 0.78 | -0.156 |
| **Memory** |  |  |  |  |  |  |  |
| HVLT-R | 48 | 25.7 (6.4) | 50 | 25.9 (5.3) | -0.66 (-4.92 to 3.61) | 0.72 | -0.154 |
| HVLT-delayed recall trial | 48 | 9.6 (3.8) | 50 | 8.8 (2.4) | 0.77 (-1.48 to 3.03) | 0.20 | 0.264 |
| **SERS** | 60 | 0.5 (1.2) | 63 | 0.4 (1.2) | -0.14 (-0.95 to 0.67) | 0.76 | -0.158 |

a, estimated OR and the 95%CI were calculated in logistic LMM; b, Effect sizes were calculated as NNT; HAMD-17=Hamilton Depression Rating Scale 17-item version; HAMA=Hamilton Anxiety Rating Scale; PHQ-9=9-item Patient Health Questionnaire-9; GAD-7=7-tiem Generalized Anxiety Disorder Scale; CGI-S=Clinical Global Impression Severity Scale; SHAPS=Snaith Hamilton Anhedonia Pleasure Scale AIS= Athens Insomnia Scale; SDS= Sheehan Disability Scale; EQ-5D-3L=EuroQol Five Dimensions Questionnaire Three-Level; DSB=Digit Span Backward Test; DSST=Digital Symbol Substitution test; TMT-A=Trail–Making Part A task; TMT-B= Trail–Making Part B test; DSF=Digit Span Forward test; HVLT=Hopkins Verbal Learning Test; SERS= Side Effects Rating Scale.

**Table 7. The impact of recruitment assessment methods on the augmenting antidepressant treatment with agomelatine**

| **Variables** | **Agomelatine + SSRI or SNRI** | | **Placebo + SSRI or SNRI** | | **Comparison** | | |
| --- | --- | --- | --- | --- | --- | --- | --- |
|  | No | Mean (SD) | No | Mean (SD) | Adjusted difference in means (95% CI) | P value | Effect size (Cohen’s d) |
| **Primary outcome** | | | | | | | |
| HAMD-17 at 8 week | 60 | 7.2 (5.2) | 63 | 7.4 (6.3) | -0.12 (-5.70 to 5.46) | 0.90 | -0.022 |
| **Secondary outcomes** | | | | | | | |
| remission | 60 | 30 (50.0%) | 63 | 33 (52.3%) | 0.88 (0.42 to 1.85) ^a^ | 0.74 | 41 ^b^ |
| response | 60 | 36 (60.0%) | 63 | 41 (65.2%) | 0.85 (0.40 to 1.80) ^a^ | 0.98 | 20 ^b^ |
| HAMD-17 at 2 week | 60 | 11.7 (4.7) | 63 | 12.4 (6.8) | -0.20 (-5.71 to 5.32) | 0.83 | -0.038 |
| HAMD-17 at 4 week | 60 | 8.5 (5.2) | 63 | 9.1 (5.6) | -0.27 (-5.65 to 5.11) | 0.76 | -0.057 |
| PHQ-9 | 60 | 7.3 (4.8) | 63 | 7.0 (6.2) | 0.39 (-4.32 to 5.1) | 0.67 | 0.077 |
| HAMA | 60 | 7.0 (6.0) | 63 | 6.8 (6.9) | 0.22 (-4.85 to 5.29) | 0.83 | 0.039 |
| GAD-7 | 60 | 4.8 (4.1) | 63 | 3.2 (3.7) | 1.32 (-1.74 to 4.38) | 0.06 | 0.351 |
| AIS | 60 | 4.7 (3.8) | 63 | 4.8 (5.2) | -0.02 (-3.88 to 3.84) | 0.98 | -0.004 |
| CGI severity | 60 | 2.5 (1.4) | 63 | 2.3 (1.5) | 0.18 (-1.23 to 1.59) | 0.43 | 0.146 |
| SHAPS | 60 | 29.2 (8.3) | 63 | 29.1 (8.5) | 0.87 (-6.47 to 8.21) | 0.51 | 0.122 |
| SDS | 56 | 2.6 (2.1) | 56 | 2.3 (2.3) | 0.36 (-1.51 to 2.22) | 0.36 | 0.176 |
| **Executive function** |  |  |  |  |  |  |  |
| DSB | 51 | 6.9 (2.2) | 48 | 7.2 (1.8) | -0.09 (-1.39 to 1.21) | 0.76 | -0.063 |
| DSST | 45 | 68.9 (12.3) | 43 | 69.4 (14.2) | -2.51 (-11.33 to 6.31) | 0.24 | -0.257 |
| Stoop Color-Word | 49 | 52.8 (17.5) | 48 | 55.9 (18.8) | -1.61 (-19.57 to 16.34) | 0.59 | -0.113 |
| TMT-B | 42 | 45.8 (17.6) | 45 | 47.7 (21.6) | -2.43 (-26.67 to 21.81) | 0.52 | -0.142 |
| **Attention** |  |  |  |  |  |  |  |
| DSF | 50 | 9.0 (1.6) | 48 | 9.3 (1.8) | 0.03 (-0.97 to 1.02) | 0.91 | 0.024 |
| Stroop-Word | 49 | 94.1 (16.6) | 48 | 96.8 (17.1) | -2.88 (-18.97 to 13.22) | 0.39 | -0.176 |
| **Processing speed** |  |  |  |  |  |  |  |
| TMT-A | 42 | 21.6 (6.6) | 45 | 21.3 (7.3) | 1.56 (-4.74 to 7.87) | 0.26 | 0.249 |
| Stroop-Color | 49 | 80.8 (18.9) | 48 | 85.5 (20.0) | -5.51 (-23.33 to 12.32) | 0.10 | -0.344 |
| **Memory** |  |  |  |  |  |  |  |
| HVLT-R | 48 | 25.7 (6.4) | 50 | 25.9 (5.3) | -0.36 (-5.29 to 4.58) | 0.69 | -0.083 |
| HVLT-delayed recall trial | 48 | 9.6 (3.8) | 50 | 8.8 (2.4) | 0.77 (-2.18 to 3.72) | 0.20 | 0.264 |
| **SERS** | 60 | 0.5 (1.2) | 63 | 0.4 (1.2) | -0.01 (-0.8 to 0.78) | 0.97 | -0.007 |

a, estimated OR and the 95%CI were calculated in logistic LMM; b, Effect sizes were calculated as NNT; HAMD-17=Hamilton Depression Rating Scale 17-item version; HAMA=Hamilton Anxiety Rating Scale; PHQ-9=9-item Patient Health Questionnaire-9; GAD-7=7-tiem Generalized Anxiety Disorder Scale; CGI-S=Clinical Global Impression Severity Scale; SHAPS=Snaith Hamilton Anhedonia Pleasure Scale AIS= Athens Insomnia Scale; SDS= Sheehan Disability Scale; EQ-5D-3L=EuroQol Five Dimensions Questionnaire Three-Level; DSB=Digit Span Backward Test; DSST=Digital Symbol Substitution test; TMT-A=Trail–Making Part A task; TMT-B= Trail–Making Part B test; DSF=Digit Span Forward test; HVLT=Hopkins Verbal Learning Test; SERS= Side Effects Rating Scale.

**Table 8. The impact of different dose of agomelatine (25 mg/50 mg) on the augmenting antidepressant treatment with Agomelatine**

| **Variables** | **Agomelatine + SSRI or SNRI** | | **Placebo + SSRI or SNRI** | | **Comparison** | | |
| --- | --- | --- | --- | --- | --- | --- | --- |
|  | No | Mean (SD) | No | Mean (SD) | Adjusted difference in means (95% CI) | P value | Effect size (Cohen’s d) |
| **Primary outcome** | | | | | | | |
| HAMD-17 at 8 week | 60 | 7.2 (5.2) | 63 | 7.4 (6.3) | 0.30 (-5.33 to 5.93) | 0.89 | 0.056 |
| **Secondary outcomes** | | | | | | | |
| remission | 60 | 30 (50.0%) | 63 | 33 (52.3%) | 0.88 (0.42 to 1.85) ^a^ | 0.74 | 41 ^b^ |
| response | 60 | 36 (60.0%) | 63 | 41 (65.2%) | 0.85 (0.40 to 1.80) ^a^ | 0.68 | 20 ^b^ |
| HAMD-17 at 2 week | 60 | 11.7 (4.7) | 63 | 12.4 (6.8) | 0.03 (-5.75 to 5.81) | 0.98 | 0.006 |
| HAMD-17 at 4 week | 60 | 8.5 (5.2) | 63 | 9.1 (5.6) | -0.15 (-5.58 to 5.28) | 0.89 | -0.031 |
| PHQ-9 | 60 | 7.3 (4.8) | 63 | 7.0 (6.2) | 0.78 (-4.25 to 5.82) | 0.68 | 0.157 |
| HAMA | 60 | 7.0 (6.0) | 63 | 6.8 (6.9) | 1.30 (-5.18 to 7.79) | 0.60 | 0.247 |
| GAD-7 | 60 | 4.8 (4.1) | 63 | 3.2 (3.7) | 1.32 (-1.97 to 4.62) | 0.06 | 0.351 |
| AIS | 60 | 4.7 (3.8) | 63 | 4.8 (5.2) | -0.02 (-4.16 to 4.12) | 0.98 | -0.004 |
| CGI severity | 60 | 2.5 (1.4) | 63 | 2.3 (1.5) | 0.20 (-1.25 to 1.65) | 0.49 | 0.164 |
| SHAPS | 60 | 29.2 (8.3) | 63 | 29.1 (8.5) | 0.93 (-6.73 to 8.59) | 0.81 | 0.130 |
| SDS | 56 | 2.6 (2.1) | 56 | 2.3 (2.3) | 0.45 (-1.64 to 2.53) | 0.30 | 0.223 |
| **Executive function** |  |  |  |  |  |  |  |
| DSB | 51 | 6.9 (2.2) | 48 | 7.2 (1.8) | -0.09 (-1.90 to 1.72) | 0.76 | -0.063 |
| DSST | 45 | 68.9 (12.3) | 43 | 69.4 (14.2) | -2.47 (-11.96 to 7.02) | 0.25 | -0.251 |
| Stoop Color-Word | 49 | 52.8 (17.5) | 48 | 55.9 (18.8) | -1.60 (-22.96 to 19.77) | 0.91 | -0.111 |
| TMT-B | 42 | 45.8 (17.6) | 45 | 47.7 (21.6) | 4.30 (-23.71 to 32.32) | 0.76 | 0.255 |
| **Attention** |  |  |  |  |  |  |  |
| DSF | 50 | 9.0 (1.6) | 48 | 9.3 (1.8) | -0.01 (-1.41 to 1.39) | 0.98 | -0.009 |
| Stroop-Word | 49 | 94.1 (16.6) | 48 | 96.8 (17.1) | -2.88 (-23.49 to 17.74) | 0.39 | -0.176 |
| **Processing speed** |  |  |  |  |  |  |  |
| TMT-A | 42 | 21.6 (6.6) | 45 | 21.3 (7.3) | 1.59 (-6.30 to 9.48) | 0.26 | 0.253 |
| Stroop-Color | 49 | 80.8 (18.9) | 48 | 85.5 (20.0) | -5.36 (-26.99 to 16.27) | 0.11 | -0.332 |
| **Memory** |  |  |  |  |  |  |  |
| HVLT-R | 48 | 25.7 (6.4) | 50 | 25.9 (5.3) | 0.19 (-5.56 to 5.94) | 0.92 | 0.045 |
| HVLT-delayed recall trial | 48 | 9.6 (3.8) | 50 | 8.8 (2.4) | 0.79 (-2.82 to 4.40) | 0.19 | 0.270 |
| **SERS** | 60 | 0.5 (1.2) | 63 | 0.4 (1.2) | -0.05 (-0.87 to 0.78) | 0.86 | -0.049 |

a, estimated OR and the 95%CI were calculated in logistic LMM; b, Effect sizes were calculated as NNT; HAMD-17=Hamilton Depression Rating Scale 17-item version; HAMA=Hamilton Anxiety Rating Scale; PHQ-9=9-item Patient Health Questionnaire-9; GAD-7=7-tiem Generalized Anxiety Disorder Scale; CGI-S=Clinical Global Impression Severity Scale; SHAPS=Snaith Hamilton Anhedonia Pleasure Scale AIS= Athens Insomnia Scale; SDS= Sheehan Disability Scale; EQ-5D-3L=EuroQol Five Dimensions Questionnaire Three-Level; DSB=Digit Span Backward Test; DSST=Digital Symbol Substitution test; TMT-A=Trail–Making Part A task; TMT-B= Trail–Making Part B test; DSF=Digit Span Forward test; HVLT=Hopkins Verbal Learning Test; SERS= Side Effects Rating Scale.

**Table 9. The impact of different antidepressants (dose equivalents to fluoxetine 20 mg ) on the effect of augmenting antidepressant treatment with agomelatine.**

| **Variables** | **Agomelatine + SSRI or SNRI** | | **Placebo + SSRI or SNRI** | | **Comparison** | | |
| --- | --- | --- | --- | --- | --- | --- | --- |
|  | No | Mean (SD) | No | Mean (SD) | Adjusted difference in means (95% CI) | P value | Effect size (Cohen’s d) |
| **Primary outcome** | | | | | | | |
| **HAMD-17 at 8 week** | 43 | 7.5 (5.1) | 54 | 6.9 (6.1) | 0.99 ( -2.79 to 4.78 ) | 0.37 | 0.188 |
| **Secondary outcomes** | | | | | | | |
| **remission** | 43 | 19 (44.2%) | 54 | 32 (59.3%) | 0.51 (0.19 to1.37) a | 0.18 | 7 ^b^ |
| **response** | 43 | 26 (60.5%) | 54 | 41 (75.9) | 0.48 (0.20 to 1.14) a | 0.18 | 7 ^b^ |
| **HAMD-17 at 2 week** | 43 | 12.4 (4.7) | 54 | 12.7 (6.9) | 0.30 (-3.86 to 4.46 ) | 0.79 | 0.057 |
| **HAMD-17 at 4 week** | 43 | 8.9 (5.5) | 54 | 9.4 (5.3) | -0.08 (-4.21 to 4.06 ) | 0.95 | -0.017 |
| **PHQ-9** | 43 | 7.6 (4.4) | 54 | 6.6 (6.1) | 1.86 (-1.93 to 5.66 ) | 0.23 | 0.406 |
| **HAMA** | 43 | 7.6 (6.2) | 54 | 6.1 (6.5) | 1.63 (-1.88 to 5.14 ) | 0.19 | 0.313 |
| **GAD-7** | 43 | 5.3 (4.0) | 54 | 3.0 (3.4) | 2.15 (-0.01 to 4.31 ) | 0.06 | 0.653 |
| **AIS** | 43 | 5.0 (4.0) | 54 | 4.5 (4.8) | 0.87 (-2.04 to 3.77 ) | 0.42 | 0.208 |
| **CGI severity** | 43 | 2.7 (1.4) | 54 | 2.3 (1.4) | 0.36 (-0.72 to 1.45 ) | 0.16 | 0.296 |
| **SHAPS** | 43 | 29.7 (7.6) | 54 | 28.6 (8.5) | 1.58 (-4.31 to 7.47 ) | 0.36 | 0.240 |
| **SDS** | 43 | 2.7 (2.0) | 54 | 1.9 (2.1) | 0.94 (0.39 to 2.28 ) | **0.02** | 0.512 |
| **Executive function** |  |  |  |  |  |  |  |
| **DSB** | 43 | 6.8 (2.4) | 54 | 7.2 (1.7) | 0.11 (-1.00 to 1.21 ) | 0.77 | 0.078 |
| **DSST** | 43 | 69.1 (12.8) | 54 | 69.5 (15.4) | -4.73 (-13.04 to 3.57 ) | 0.21 | -0.484 |
| **Stoop Color-Word** | 43 | 49.2 (15.9) | 54 | 54.8 (19.6) | -3.85 (-17.56 to 9.86 ) | 0.44 | -0.320 |
| **TMT-B** | 43 | 45.9 (17.5) | 54 | 49.5 (23.1) | -8.98 (-29.57 to 11.60) | 0.156 | -0.578 |
| **Attention** |  |  |  |  |  |  |  |
| **DSF** | 43 | 8.7 (1.7) | 54 | 9.3 (1.8) | -0.04 (-0.79 to 0.71 ) | 0.911 | -0.035 |
| **Stroop-Word** | 43 | 95.5 (12.8) | 54 | 95.5 (17.6) | 0.11 (-9.40 to 9.62 ) | 0.973 | 0.008 |
| **Processing speed** |  |  |  |  |  |  |  |
| **TMT-A** | 43 | 21.6 (6.8) | 54 | 21.4 (7.8) | 2.16 (-2.66 to 6.97 ) | 0.397 | 0.383 |
| **Stroop-Color** | 43 | 79.1 (16.7) | 54 | 84.3 (20.8) | -2.57 (-14.92 to 9.77 ) | 0.527 | -0.189 |
| **Memory** |  |  |  |  |  |  |  |
| **HVLT-R** | 43 | 26.1 (6.8) | 54 | 22.0 (9.9) | 3.49 (-3.15 to 10.13 ) | 0.080 | 0.487 |
| **HVLT-delayed recall trial** | 43 | 9.7 (4.1) | 54 | 8.5 (2.5) | 1.53 (-0.94 to 4.00 ) | 0.173 | 0.581 |
| **SERS** | 43 | 0.5 (1.3) | 54 | 0.4 (1.2) | 0.14 (-0.80 to 1.08 ) | 0.743 | 0.148 |

a, estimated OR and the 95%CI were calculated in logistic LMM; b, Effect sizes were calculated as NNT; HAMD-17=Hamilton Depression Rating Scale 17-item version; HAMA=Hamilton Anxiety Rating Scale; PHQ-9=9-item Patient Health Questionnaire-9; GAD-7=7-tiem Generalized Anxiety Disorder Scale; CGI-S=Clinical Global Impression Severity Scale; SHAPS=Snaith Hamilton Anhedonia Pleasure Scale AIS= Athens Insomnia Scale; SDS= Sheehan Disability Scale; EQ-5D-3L=EuroQol Five Dimensions Questionnaire Three-Level; DSB=Digit Span Backward Test; DSST=Digital Symbol Substitution test; TMT-A=Trail–Making Part A task; TMT-B= Trail–Making Part B test; DSF=Digit Span Forward test; HVLT=Hopkins Verbal Learning Test; SERS= Side Effects Rating Scale

**Table 10. The efficacy of augmenting antidepressant treatment with agomelatine without employing LOCF**

| **Variables** | **Agomelatine + SSRI or SNRI** | | **Placebo + SSRI or SNRI** | | **Comparison** | | |
| --- | --- | --- | --- | --- | --- | --- | --- |
|  | No | Mean (SD) | No | Mean (SD) | Adjusted difference in means (95% CI) | P value | Effect size (Cohen’s d) |
| **Primary outcome** | | | | | | | |
| HAMD-17 at 8 week | 56 | 6.9 (5.2) | 55 | 7.2 (6.5) | -0.34 (-4.37 to 3.68) | 0.74 | -0.063 |
| **Secondary outcomes** | | | | | | | |
| remission | 56 | 29 (51.8%) | 55 | 30 (54.5%) | 0.89 (0.41 to 1.93) ^a^ | 0.76 | 36 ^b^ |
| response | 56 | 35 (62.5%) | 55 | 36 (65.5%) | 0.91 (0.41 to 1.99) ^a^ | 0.81 | 20 ^b^ |
| HAMD-17 at 2 week | 56 | 11.8 (4.7) | 55 | 12.4 (6.8) | -0.23 (-3.99 to 3.54) | 0.82 | -0.044 |
| HAMD-17 at 4 week | 55 | 8.3 (5.2) | 55 | 9.1 (5.6) | -0.62 (-4.82 to 3.58) | 0.50 | -0.132 |
| PHQ-9 | 56 | 7.3 (4.8) | 55 | 7.0 (6.2) | 0.35 (-2.89 to 3.58) | 0.72 | 0.068 |
| HAMA | 56 | 6.6 (6.0) | 55 | 6.8 (6.9) | 0.06 (-3.85 to 3.97) | 096 | 0.010 |
| GAD-7 | 55 | 4.6 (4.2) | 55 | 3.2 (3.7) | 0.94 (-1.18 to 3.05) | 0.20 | 0.242 |
| AIS | 55 | 4.9 (3.7) | 55 | 4.8 (5.2) | -0.34 (-3.39 to 2.71) | 0.68 | -0.079 |
| CGI severity | 54 | 2.5 (1.4) | 54 | 2.3 (1.5) | 0.10 (-5.60 to 5.81) | 0.44 | 0.015 |
| SHAPS | 56 | 29.2 (8.3) | 55 | 29.1 (8.5) | 0.82 (-5.28 to 6.92) | 0.56 | 0.113 |
| SDS | 56 | 2.6 (2.3) | 55 | 2.6 (2.1) | 0.33 (-0.93 to 1.59) | 0.40 | 0.163 |
| **Executive function** |  |  |  |  |  |  |  |
| DSB | 50 | 6.9 (2.2) | 46 | 7.3 (1.8) | -0.16 (-0.9 to 0.58) | 0.58 | -0.115 |
| DSST | 45 | 68.5 (12.3) | 42 | 69.4 (14.2) | -1.56 (-7.65 to 4.54) | 0.42 | -0.175 |
| Stoop Color-Word | 46 | 52.4 (17.3) | 50 | 55.9 (19) | -1.49 (-14.78 to 11.80) | 0.62 | -0.104 |
| TMT-B | 42 | 46.4 (17.7) | 44 | 47.7 (21.6) | -2.04 (-19.74 to 15.66) | 0.59 | -0.118 |
| **Attention** |  |  |  |  |  |  |  |
| DSF | 50 | 9.1 (2.2) | 46 | 9.2 (1.8) | 0.01 (-0.55 to 0.57) | 0.97 | 0.009 |
| Stroop-Word | 46 | 93.9 (16.5) | 50 | 96.9 (17.3) | -3.20 (-11.82 to 5.42) | 0.34 | -0.196 |
| **Processing speed** |  |  |  |  |  |  |  |
| TMT-A | 42 | 21.5 (6.5) | 44 | 21.3 (7.3) | 1.10 (-3.34 to 5.54) | 0.22 | 0.186 |
| Stroop-Color | 46 | 80.4 (19.0) | 50 | 85 (19.9) | -5.30 (-16.12 to 5.51) | 0.12 | -0.327 |
| **Memory** |  |  |  |  |  |  |  |
| HVLT-R | 47 | 26.0 (5.4) | 49 | 25.9 (5.3) | -0.57 (-3.12 to 1.98) | 0.65 | -0.130 |
| HVLT-delayed recall trial | 47 | 9.1 (2.2) | 49 | 8.8 (2.4) | 0.78 (-0.72 to 2.28) | 0.20 | 0.266 |
| **SERS** | 52 | 0.49 (1.2) | 51 | 0.37 (1.6) | -0.01 (-0.55 to 0.52) | 0.95 | -0.014 |

a, estimated OR and the 95%CI were calculated in logistic LMM; b, Effect sizes were calculated as NNT; HAMD-17=Hamilton Depression Rating Scale 17-item version; HAMA=Hamilton Anxiety Rating Scale; PHQ-9=9-item Patient Health Questionnaire-9; GAD-7=7-tiem Generalized Anxiety Disorder Scale; CGI-S=Clinical Global Impression Severity Scale; SHAPS=Snaith Hamilton Anhedonia Pleasure Scale AIS= Athens Insomnia Scale; SDS= Sheehan Disability Scale; EQ-5D-3L=EuroQol Five Dimensions Questionnaire Three-Level; DSB=Digit Span Backward Test; DSST=Digital Symbol Substitution test; TMT-A=Trail–Making Part A task; TMT-B= Trail–Making Part B test; DSF=Digit Span Forward test; HVLT=Hopkins Verbal Learning Test; SERS= Side Effects Rating Scale; LOCF =last observation carried forward procedure.

**Table 11. A comparison of adverse event between the two treatment groups over the course of 8 weeks**

|  | **All Participants** | **Agomelatine + SSRI or SNRI** | **Placebo + SSRI or SNRI** | **P-value** |
| --- | --- | --- | --- | --- |
| AEs | 25.2% (31/123) | 23.3% (14/60) | 26.9% (17/63) | 0.796 |
| SAEs | 0% (0/60) | 0% (0/63) | 0% (0/123) | 1 |

AEs=adverse events; SAEs=serious adverse events; SSRI=selective serotonin reuptake inhibitor; SNRI=serotonin-noradrenaline reuptake inhibitor

**Table 12. A comparison of abnormal transaminase levels between the two treatment groups over the course of 8 weeks**

|  | **All Participants** | **Agomelatine + SSRI or SNRI** | **Placebo + SSRI or SNRI** | **P** |
| --- | --- | --- | --- | --- |
| **Abnormal transaminase levels** | 2.43% (3/123) | 1.67% (1/60) | 3.17% (2/63) | 1 |

SSRI=selective serotonin reuptake inhibitor; SNRI=serotonin-noradrenaline reuptake inhibitor; P value obtained by Fisher's Exact Test

**Table 13. A comparison of adverse event between the two treatment groups at week 8**

| **Adverse Event** | **Agomelatine + SSRI or SNRI**  **N** | **Agomelatine + SSRI or SNRI**  **%** | **Placebo + SSRI or SNRI N** | **Placebo + SSRI or SNRI %** | **P-value** |
| --- | --- | --- | --- | --- | --- |
| Fatigue | 6 | 10.00% | 7 | 11.11% | 1.00 |
| Headache | 3 | 5.00% | 2 | 3.17% | 0.67 |
| Insomnia | 5 | 8.33% | 7 | 11.11% | 0.83 |
| Dizziness | 4 | 6.67% | 3 | 4.76% | 0.71 |
| Orthostatic hypotension | 2 | 3.33% | 1 | 1.59% | 0.61 |
| Palpitations | 5 | 8.33% | 3 | 4.76% | 0.66 |
| Tremor | 1 | 1.67% | 0 | 0.00% | 0.49 |
| Sweating | 3 | 5.00% | 3 | 4.76% | 1.00 |
| Dry mouth | 5 | 8.33% | 4 | 6.35% | 0.94 |
| Constipation | 4 | 6.67% | 5 | 7.94% | 1.00 |
| urinary difficulties | 1 | 1.67% | 1 | 1.59% | 1.00 |
| Drowsy | 1 | 1.67% | 3 | 4.76% | 0.62 |
| Sexual dysfunction | 3 | 5.00% | 1 | 1.59% | 0.36 |

P-values were obtained from Chi-Square test and Fisher’s test; AE=adverse events, SSRI=selective serotonin reuptake inhibitor; SNRI=serotonin-noradrenaline reuptake inhibitor
